# Molecular Characterization and Its Clinical Application of *GNAS* Variants in Intramuscular Myxoma

**DOI:** 10.1002/cam4.71751

**Published:** 2026-03-29

**Authors:** Munehisa Kito, Shohei Shigeto, Mai Iwaya, Tomomi Yamaguchi, Masanori Okamoto, Atsushi Tanaka, Akira Takazawa, Hirokazu Ideta, Kaoru Aoki, Hiromasa Hasegawa, Kenji Sano, Tomomi Fujikawa, Tomoki Kosho, Jun Takahashi

**Affiliations:** ^1^ Department of Orthopaedic Surgery Shinshu University School of Medicine Nagano Japan; ^2^ Department of Laboratory Medicine Shinshu University Hospital Nagano Japan; ^3^ Department of Medical Genetics Shinshu University School of Medicine Nagano Japan; ^4^ Center for Medical Genetics Shinshu University Hospital Nagano Japan; ^5^ Division of Clinical Sequencing Shinshu University School of Medicine Nagano Japan; ^6^ Department of Oral Pathology/Forensic Odontology Aichi Gakuin University School of Dentistry Nagoya Aichi Japan; ^7^ Department of Pathology Iida Municipal Hospital Nagano Japan; ^8^ Research Center for Supports to Advanced Science Shinshu University Nagano Japan; ^9^ Biobank Shinshu Shinshu University Hospital Nagano Japan

**Keywords:** fragment signal analysis, *GNAS*, intramuscular myxoma, low‐grade myxofibrosarcoma, NGS, PNA

## Abstract

**Background:**

Intramuscular myxoma (IM) is a benign tumor that harbors *GNAS* missense variants. Distinguishing IM from low‐grade myxofibrosarcoma (LGMFS) is challenging due to similarities in imaging and histological features. While molecular analysis aids differentiation, the low number of tumor cells available for DNA extraction necessitates highly accurate detection methods for variant identification. The aim of this study was to delineate molecular characteristics using next‐generation sequencing (NGS) and to propose an optimal screening method in a clinical setting to differentiate IM from LGMFS.

**Materials:**

Ten IM cases and nine LGMFS cases with extractable DNA from FFPE samples were recruited.

**Results:**

A custom NGS panel‐based analysis for *GNAS* revealed R201C/R201H variants in resected samples of eight cases with IM and in none of the eight cases, excluding one with insufficient sample quality, with LGMFS, and in biopsy samples of both available cases with IM. Additionally, various sequence alterations were detected irrespective of the clinical status (IM, LGMFS, and normal tissue) or sample conditions (resected, biopsy, and relapsed). The detection rate of *GNAS*‐positive IM using other methods (PCR‐direct sequencing, fragment signal analysis using restriction digestion and capillary electrophoresis after PCR combined with peptide nucleic acid (PNA) clamping, and PCR‐direct sequencing combined with PNA clamping) was calculated as 0.42, 0.83, and 0.75, respectively, while the detection rate for GNAS‐negative LGMFS was 1.

**Conclusion:**

These findings suggest that fragment signal analysis is a reasonable diagnostic approach for differentiating IM from LGMFS. However, it is important to recognize that the absence of GNAS R201C/R201H variants does not rule out IM.

## Graphical abstract

This graphical abstract illustrates the workflow of fragment signal analysis for detecting GNAS codon 201 variants, combining PNA clamping PCR, restriction digestion, and capillary electrophoresis to differentiate wild‐type and variant alleles.

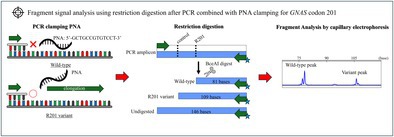

## 1 Introduction

Intramuscular myxoma (IM) is a benign mesenchymal tumor. The tumor is composed of spindle‐shaped cells and features abundant mucus and hypovascular stroma. The age of onset is middle‐aged and older, and most cases occur within the muscles of the extremities; however, the tumor may also occur subcutaneously [1, 2, 3]. On imaging, IM shows signal changes reflecting the abundance of myxoid stroma and is characterized by little contrast enhancement due to poor blood flow [4, 5, 6]. Simple tumor resection rarely causes recurrence [7, 8].

Differentiating between IM and myxoid malignant soft tissue tumors, such as myxofibrosarcoma, low‐grade fibromyxoid sarcoma, myxoid liposarcoma, and extraskeletal myxoid chondrosarcoma, can be challenging. However, tumors other than myxofibrosarcoma can be differentiated from IM by specific immunostaining and searching for fusion genes [9, 10].

Myxofibrosarcoma is a relatively common malignant soft tissue tumor that occurs in the extremities of middle‐aged and elderly individuals. Similar to IM, imaging shows signal changes reflecting the abundance of mucus stroma, and the contrast effects vary depending on the case. Histopathologically, the tumor is similar to that of IM, as it is composed of an abundance of myxoid stroma and spindle‐shaped cells; however, if the tumor is highly malignant, it may show high nuclear staining, atypia, and mitosis compared to IM [11, 12]. Differentiation from IM is difficult due to the lack of such findings in cases of low malignancy [11]. The conventional treatment is wide excision, and adjuvant chemotherapy and radiotherapy may also be used [13]. Because the treatment strategies for IM and low‐grade myxofibrosarcoma (LGMFS) are different, a tool is needed to differentiate these two types of tumors.

Missense variants in *GNAS* have been identified in IM [14, 15, 16]. *GNAS* is located on chromosome 20q13.2‐q13.3, and it contains 13 exons. This gene encodes a component of the GTP‐binding protein (G protein) and is involved in cell signal transduction. G proteins are composed of a trimer of α, β, and γ and are activated by G protein‐coupled receptors. *GNAS* is an α subunit (GαS) that binds to GDP in its inactive form but binds to GTP upon activation and differentiates it from the βγ dimer. Activated *GNAS* increases the GαS‐cAMP concentration by activating adenylate cyclase and cAMP‐dependent protein kinase [17]. These variants included missense variants at codon 201 of exon 8 (R201C, R201H, R201S, R201L, and R201P) and codon 227 of exon 9 (Q227E), most of which were R201C and R201H [16, 18, 19]. R201C and R201H have been reported to be useful for differentiating IM from LGMFS, in which R201 variants were not observed [16].

However, an optimal genetic screening system for *GNAS* variants in IM has not been established because of the small number of tumor cells in the tissue of IM and the low frequency of variant alleles [15]. In direct sequencing analysis, the positive rates for *GNAS* variants were not high, ranging from 29% to 50% [15, 16, 20]. Recent next‐generation sequencing (NGS) analyses showed higher positive rates (88%, 92%) [18, 21].

Peptide nucleic acid (PNA) clamping is a method that increases the detection sensitivity of direct sequencing [22, 23, 24]. PNA is a DNA mimic in which the deoxyribose phosphate backbone is replaced with a 2‐aminoethylglycine backbone. In this method, the PNA primer binds to the normal base sequence, inhibiting the PCR amplification of the wild‐type allele. Consequently, variant alleles can be emphasized and amplified. Direct sequencing analysis combined with PNA clamping has been reported to identify variants in 87.5% of cases in peripheral blood cells of fibrous dysplasia/McCune‐Albright syndrome, with the same *GNAS* variants as IM, which had been difficult to identify [25].

Fragment signal analysis by restriction enzyme digestion and capillary electrophoresis was used to search for missense variants. This method was used to detect missense variants at codon 835 in *FLT3* in acute myeloid leukemia [26]. PCR products amplified with fluorescently labeled primers were treated with restriction enzyme (EcoRV) to cleave only the amplified product of the wild‐type allele, and the wild‐type and mutant alleles were ultimately separated by fragment signal analysis using capillary electrophoresis. This has been commercialized as a companion diagnostic product (LeukoStrat CDx *FLT3* Mutation Assay). Fragment signal analysis using capillary electrophoresis has superior resolution compared to fragment analysis using gel electrophoresis and allows semi‐quantitative determination. Because direct sequencing is not required, the analysis time is short, and it has the advantage of being independent of the type of missense variant in the target codon.

The purpose of this study was to establish the molecular characterization of *GNAS* variants in IM compared with LGMFS through a custom NGS panel analysis and to explore the optimal genetic screening system for *GNAS* variants in IM among four methods (NGS, PCR‐direct sequencing with/without PNA, and fragment signal analysis), which is useful for differentiating IM from LGMFS.

## 2 Materials and Methods

### 2.1 Patient Data

**FIGURE 1:**
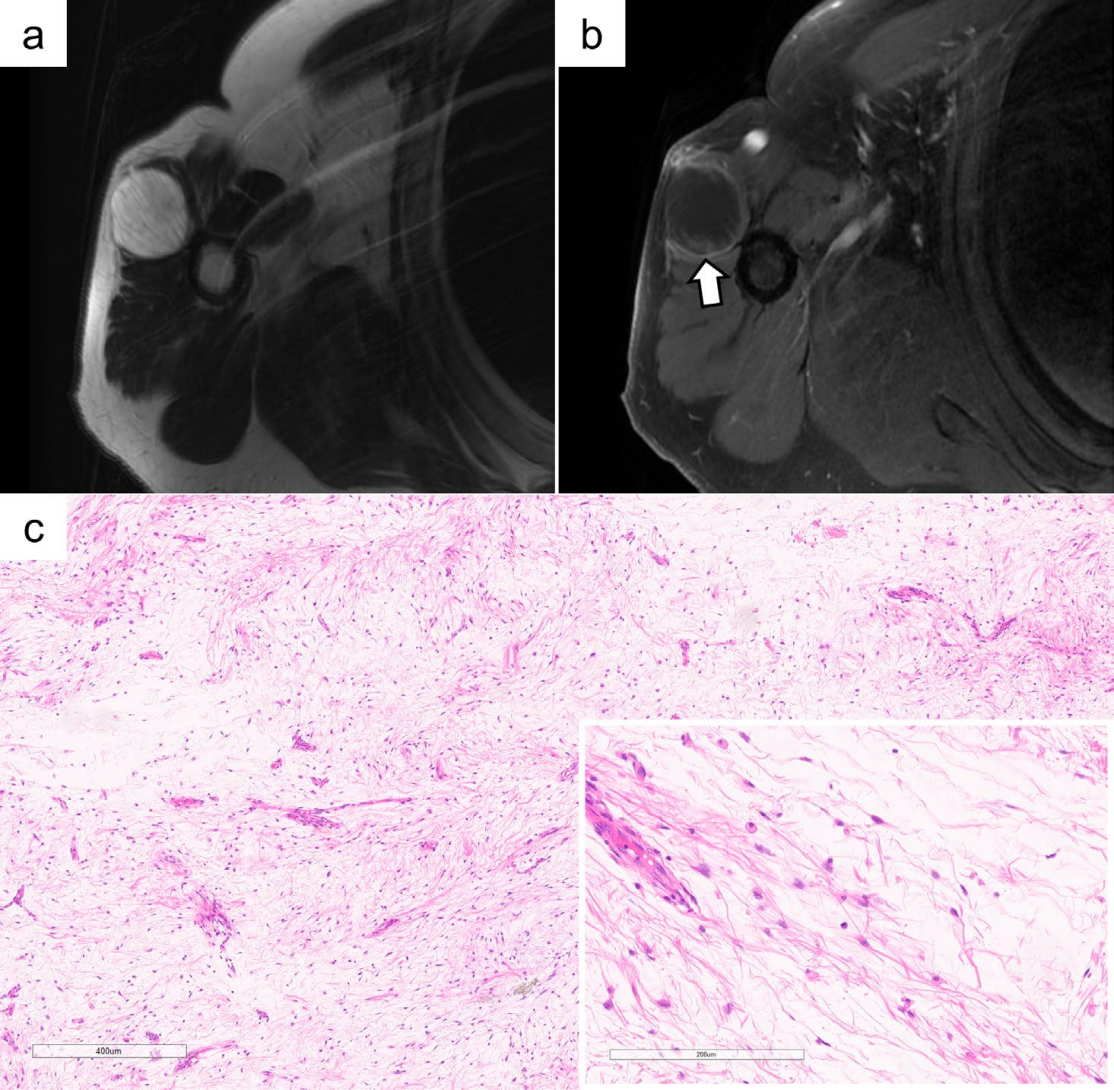
IM case (Patient #6): A high signal intensity mass is observed within the deltoid muscle on MRI T2‐weighted imaging (a). The mass is faintly enhanced in part, as indicated by the arrow (b). Histologically, focal hypercellular areas are seen against a background of fibrous stroma. Nuclear chromatin is not markedly hyperchromatic. Occasional branching capillaries are present (c).

**FIGURE 2:**
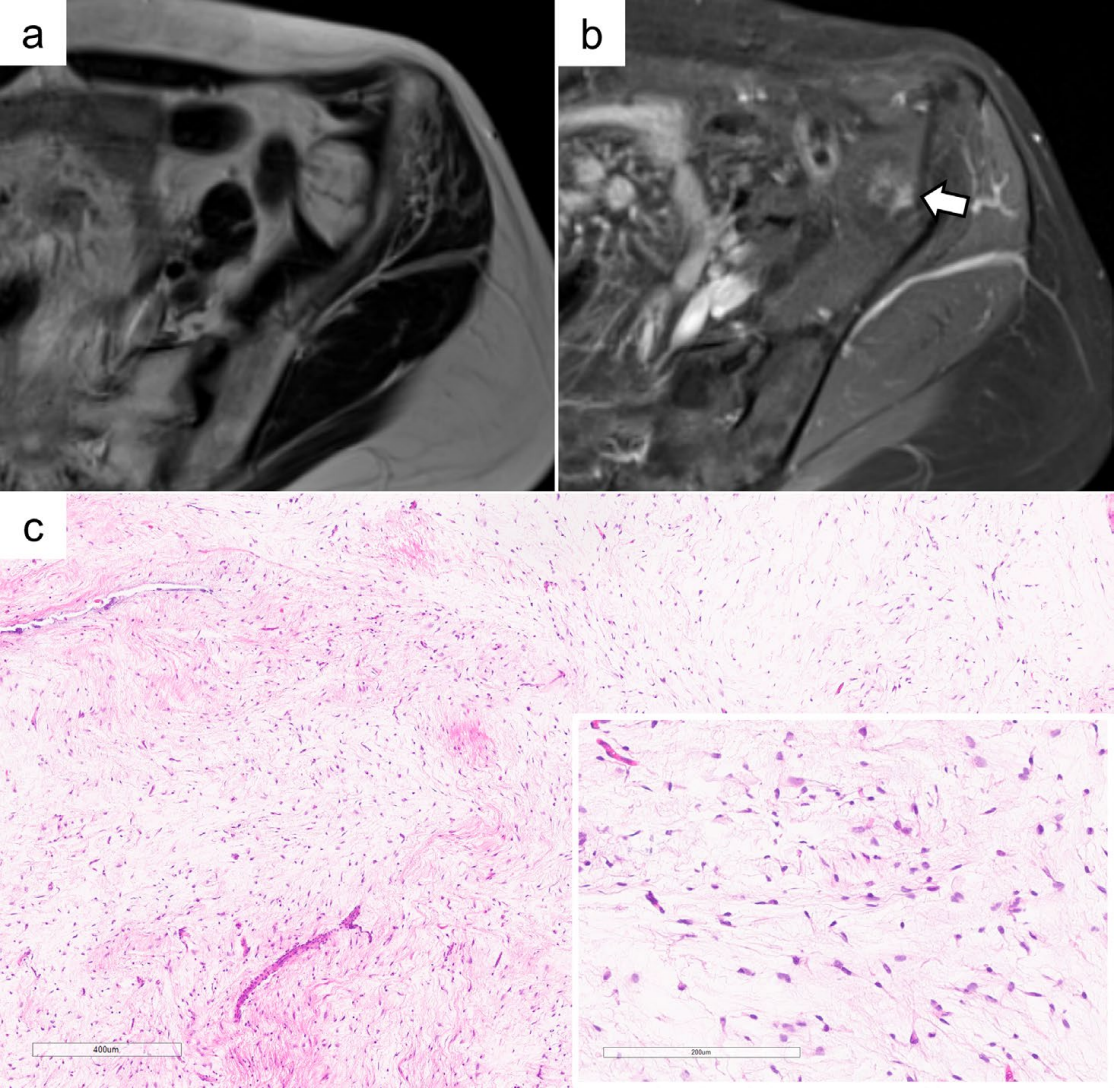
LGMFS case (Patient #18): MRI T2‐weighted imaging shows a high signal intensity mass within the iliacus muscle (a). The mass exhibits faint enhancement in part, as indicated by the arrow (b). Histologically, the findings resemble those of Figure 1; however, the tumor cells exhibit slightly larger nuclei with hyperchromasia. Occasional branching capillaries are present (c).

Between 2009 and 2020, 10 cases of IM and 9 cases of LGMFS were recruited at Shinshu University Hospital. Patients with DNA that could be extracted from FFPE were included in this study. Table 1 presents the characteristics of these cases. The median sizes of IM and LGMFS were 48.5 mm (range, 18–141 mm) and 71.0 mm (range, 38–143 mm), respectively. Surgery was performed in all cases, and the surgical methods for IM were marginal resection in 5 cases and wide resection in 5 cases, and those of LGMFS were marginal resection in 1 case and wide resection in 8 cases. In all cases in which wide resection was performed for IM, the biopsy specimens were not differentiated as benign or malignant. In addition, in LGMFS, where marginal resection was performed, the biopsy specimen was diagnosed as IM. Therefore, additional wide resection was performed postoperatively. Recurrence was observed in one case of LGMFS treated with wide resection, and no cases developed metastasis. The median follow‐up period was 26.1 months (range, 1.5–59.3 months) for IM and 64.8 months (range, 3.0–120.5 months) for LGMFS. Histopathological diagnosis was performed in accordance with the WHO Classification of Tumours/Soft Tissue and Bone Tumors, 5th edition [27]. After the start of this study, a blinded rediagnosis was performed retrospectively by three pathologists (M.I., H. H., K. S.). Rediagnosis was performed based solely on histopathological findings, except for genetic analysis. If a difference in diagnosis occurred, a discussion was held to reach consensus on the diagnosis. Representative cases of IM and LGMFS are shown in Figure 1 and Figure 2, respectively.

**TABLE 1:** Patient characteristics.

No.	Diagnosis		Gender	Age	Site	Tumor size, mm	Treatment	LR	DM
	Biopsy	Resection							
1	IM or LGMFS	IM	F	43	UE	18	ME	—	—
2	IM	IM	F	64	LE	141	ME	—	—
3	—	IM	M	52	LE	29	ME	—	—
4	IM or LGMFS	IM	M	57	UE	65	WE	—	—
5	IM or LGMFS	IM	M	71	Trunk	71	WE	—	—
6	IM or LGMFS	IM	M	62	UE	57	WE	—	—
7	—	IM	M	87	LE	18	ME	—	—
8	IM	IM	F	60	LE	23	ME	—	—
9	IM or LGMFS	IM	F	68	LE	69	WE	—	—
10	IM or LGMFS	IM	F	49	LE	40	WE	—	—
11	LGMFS	LGMFS	M	73	LE	88	WE	—	—
12	LGMFS	LGMFS	M	70	UE	35	WE	—	—
13	LGMFS	LGMFS	F	77	UE	72	WE	+	—
14	LGMFS	LGMFS	M	76	LE	143	WE	—	—
15	IM	LGMLS	F	19	Trunk	38	ME	—	—
16	LGMFS	LGMFS	F	40	LE	129	WE	—	—
17	LGMFS	LGMFS	F	70	LE	49	WE	—	—
18	IM or LGMFS	LGMFS	F	53	Trunk	37	WE	—	—
19	LGMFS	LGMFS	M	68	LE	71	WE	—	—

Abbreviations: DM, distant metastases; F, female; IM, intramuscular myxoma; LE, lower extremities; LGMFS, low‐grade myxofibrosarcoma; LR, local relapsed; M, male; ME, marginal excision; NA, not available; UE, upper extremities; WE, wide excision.

This study was approved by the institutional ethics review board of Shinshu University School of Medicine. Informed consent was obtained from all participants where possible. In cases where obtaining consent was not feasible, the study information was disclosed publicly and an opt‐out procedure was employed, in accordance with institutional and ethical guidelines.

### 2.2 DNA Extraction

Using HE‐stained slides with tumor‐rich areas marked by a pathologist, non‐stained slides of FFPE were macro‐dissected and used for DNA extraction. DNA extraction was performed using the QIAamp DNA FFPE Tissue Kit (Qiagen, Hilden, Germany), according to the manufacturer's protocol. The extracted DNA was quantified using the Qubit 1X dsDNA High Sensitivity Assay Kit (Invitrogen, Waltham, MA, USA) and stored at −20°C.

### 2.3 A Custom NGS Panel‐Based Analysis for GNAS

Targeted NGS was performed using a custom *GNAS* panel optimized for FFPE samples and for detection of hot spot variants at codon 201 while variants in all exons and exon‐intron borders can be detected. Sequencing data were processed using established pipelines.

Complete details of panel design, library preparation, sequencing platforms, bioinformatic processing, and variant annotation criteria are described in Supplementary Methods S1.

### 2.4 Genetic Screening for R201 Variants in GNAS

**FIGURE 3:**
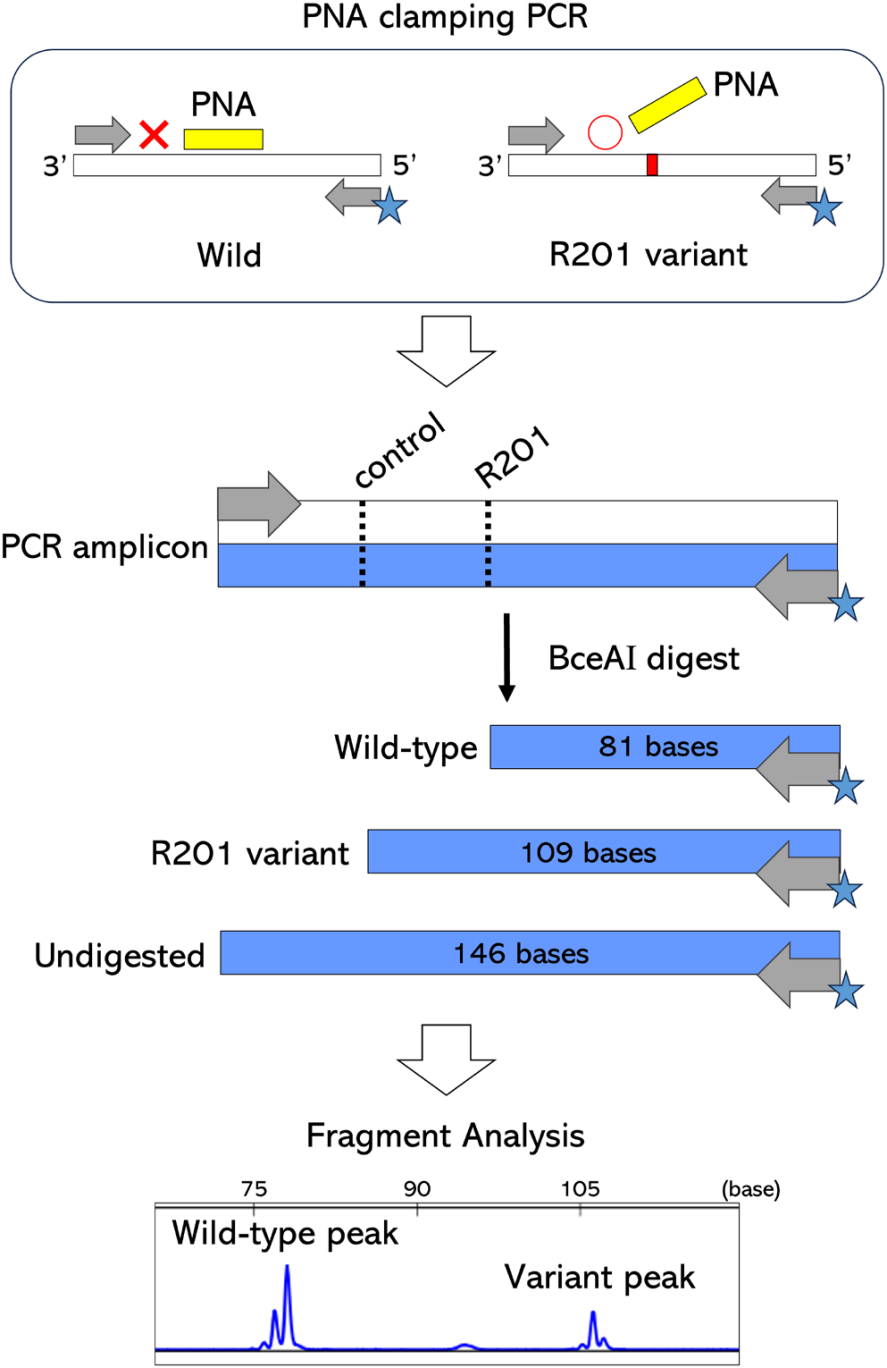
PCR reaction using the PNA (yellow) clamping to target R201 in *GNAS* is performed (reverse labeled with FAM (blue, star), forward unlabeled). After amplification with emphasis on the R201 variant allele, the PCR product is digested with BceAI. The dotted line in the PCR product shows the BceAI cut site and the recognition sequence (ACGGC). After digestion, the R201 portion of the assay yields the FAM‐labeled wild‐type product of 81 bases; the FAM‐labeled product of the R201 variant is 109 bases, and the undigested FAM‐labeled product is 146 bases.

To evaluate the major *GNAS* variants (R201C and R201H) in IM, three complementary genetic screening approaches were employed: PCR‐direct sequencing, Fragment signal analysis by restriction digestion and capillary electrophoresis after PCR combined with PNA clamping (Figure 3), and PCR‐direct sequencing analysis combined with PNA clamping.

Full experimental protocols, primer sequences, PCR conditions, restriction digestion, capillary electrophoresis, signal ratio calculation, and assay schema are provided in Supplementary Methods S2.1–S2.3.

Analytical sensitivity was assessed using plasmid‐based dilution experiments, as detailed in Supplementary Methods S2.4.

### 2.5 Statistical Analysis

To set cutoff values for allele relative values in fragment signal analysis, the Dunn test with merged ranks was used as a non‐parametric comparison with the control group. The association between the SR, measured by fragment signal analysis, and the variant allele frequency (VAF), measured by NGS, was evaluated using Spearman's rank correlation coefficient. Data were analyzed using JMP version 14.2 (SAS Institute, Cary, NC, USA), and a *P*‐value of < 0.05 was considered statistically significant.

## 3 Results

### 3.1 A Custom NGS Panel‐Based Analysis for GNAS

Table 2 presents the results of the R201C/H analysis. Among the resected tumor samples from 10 patients with IM, R201C was detected in four cases (#2, #5, #6, #9), R201H was detected in four cases (#1, #4, #8, #10), and neither R201C nor R201H was detected in two cases (#3, #7). Neither R201C nor R201H was detected among resected tumor samples of nine cases with LGMFS, in one of whom (#11, depth 0) insufficient quality of the sample was the cause. Among biopsy samples of two cases with IM, R201C was detected in one case (#2) and R201H was detected in one case (#4), similar to the same results as resected tumor samples. Neither R201C nor R201H was detected among the biopsy samples of four cases with LGMFS, in two of which (#15, depth 120; #16, depth 40), insufficient quality of the sample was the cause. Neither R201C nor R201H was detected in a resected tumor sample from one case in the relapsed state with LGMFS (#13). Neither R201C nor R201H was detected in resected normal tissue samples from one case with IM (#6) or in those 3 cases with LGMFS (#13 in the relapsed state, #15, #18).

**TABLE 2:** Comparison among screening methods for R201.

No.	Final diagnosis	Specimen	Variant	Variant analysis						
				NGS			PCR‐DS	Fragment signal analysis		PCR‐DS with PNA
					VAF	coverage of depth			SR	
1	IM	Resection	R201C	−	0.000	1114	−	+	0.42	−
			R201H	**+**	0.473	1114	−			+
2	IM	Biopsy	R201C	**+**	0.164	10,367	+	+	0.49	+
			R201H	−	0.000	10,364	−			−
		Resection	R201C	**+**	0.210	9117	+	+	0.81	+
			R201H	−	0.000	9116	−			−
3	IM	Resection	R201C	−	0.000	9567	−	−	0.04	−
			R201H	−	0.000	9569	−			−
4	IM	Biopsy	R201C	−	0.000	10,929	−	+	0.17	−
			R201H	**+**	0.017	10,926	−			−
		Resection	R201C	−	0.000	13,135	−	+	0.61	−
			R201H	**+**	0.187	13,131	−			+
5	IM	Resection	R201C	**+**	0.091	5155	−	+	0.31	+
			R201H	−	0.000	5156	−			−
6	IM	Resection	R201C	**+**	0.227	590	+	+	0.35	+
			R201H	−	0.000	590	−			−
		Normal	R201C	−	0.000	486	NA	NA	NA	NA
			R201H	−	0.000	486	NA			NA
7	IM	Resection	R201C	−	0.000	12,404	−	−	0.05	−
			R201H	−	0.000	12,404	−			−
8	IM	Resection	R201C	−	0.000	11,508	−	+	0.53	−
			R201H	**+**	0.153	11,503	−			+
9	IM	Resection	R201C	**+**	0.114	14,812	+	+	0.72	+
			R201H	−	0.000	14,808	−			−
10	IM	Resection	R201C	−	0.000	5355	−	+	0.58	−
			R201H	**+**	0.205	5350	+			+
11	LGMFS	Resection	R201C	ND	—	0	−	−	0.02	−
			R201H	ND	—	0	−			−
12	LGMFS	Resection	R201C	−	0.000	14,628	−	−	0.03	−
			R201H	−	0.000	14,622	−			−
13	LGMFS	Resection	R201C	−	0.000	2138	−	−	0.03	−
			R201H	−	0.000	2137	−			−
		Resection (relapsed)	R201C	−	0.000	12,676	−	−	0.02	−
			R201H	−	0.000	12,672	−			−
		Normal (relapsed)	R201C	−	0.000	9049	NA	NA	NA	NA
			R201H	−	0.000	9049	NA			NA
14	LGMFS	Biopsy	R201C	−	0.000	2132	−	−	0.04	−
			R201H	−	0.000	2132	−			−
		Resection	R201C	−	0.000	541	−	−	0.02	−
			R201H	−	0.000	540	−			−
15	LGMLS	Biopsy	R201C	−	0.000	120	−	−	0.02	−
			R201H	−	0.000	120	−			−
		Resection	R201C	−	0.000	4363	−	−	0.03	−
			R201H	−	0.000	4362	−			−
		Normal	R201C	−	0.000	5855	NA	NA	NA	NA
			R201H	−	0.000	5854	NA			NA
16	LGMFS	Biopsy	R201C	−	0.000	40	−	−	0.02	−
			R201H	−	0.000	40	−			−
		Resection	R201C	−	0.000	9183	−	−	0.02	−
			R201H	−	0.000	9180	−			−
17	LGMFS	Biopsy	R201C	−	0.000	3091	−	−	0.02	−
			R201H	−	0.000	3091	−			−
		Resection	R201C	−	0.000	4504	−	−	0.03	−
			R201H	−	0.000	4503	−			−
18	LGMFS	Resection	R201C	−	0.000	354	−	−	0.04	−
			R201H	−	0.000	354	−			−
		Normal	R201C	−	0.000	3741	NA	NA	NA	NA
			R201H	−	0.000	3741	NA			NA
19	LGMFS	Resection	R201C	−	0.000	11,688	−	−	0.02	−
			R201H	−	0.000	11,687	−			−

Abbreviations: fragment signal analysis, fragment signal analysis using restriction digestion and capillary electrophoresis after PCR combined with PNA clamping; IM, intramuscular myxoma; LGMFS, low‐grade myxofibrosarcoma; NA, not available; ND, not detected; NGS, next‐generation sequencing; normal, normal tissue around the tumor; PCR‐DS with PNA, PCR‐direct sequencing combined with PNA clamping; PCR‐DS, PCR‐direct sequencing; SR, signal ratio; VAF, variant allele frequency.

The results of the comprehensive variant characterization of *GNAS* are shown in Supplementary Table S1. Among the resected tumor samples from 10 cases with IM, the median number of detected variants was 1 (range, 0–13). Among the resected tumor samples from 9 cases with LGMFS, the median number of detected variants was 4 (range, 0–16). Among the biopsy samples of two cases with IM, #2 was found to have only one variant (R201C), the same as the resected tumor sample; and #4 was found to have two variants (including R201H), whereas only R201H was detected in the resected tumor sample. Among biopsy samples of five cases with LGMFS, #14 was found to have 11 variants, whereas eight variants were detected in the resected tumor sample with no overlapping variants; #15 was found to have no variant, whereas 12 variants were detected in the resected tumor sample; #16 was found to have variants due to insufficient quality of the sample; and #17 was found to have no variant, whereas four variants were detected in the resected tumor sample. Among normal tissue samples from one patient with IM and three cases with LGMFS, #6 with IM was found to have 13 variants, whereas eight variants were detected in the resected tumor sample with no overlapping variants; #13 with LGMFS was found to have no variant, whereas 16 variants were detected in the resected tumor sample; #15 with LGMFS was found to have no variant, whereas 12 variants were detected in the resected tumor sample; and #18 with LGMFS was found to have one variant, whereas six variants were detected in the resected tumor sample.

### 3.2 Common GNAS Variant (R201C/R201H) Screening

#### 3.2.1 Establishment of Fragment Signal Analysis

**FIGURE 4:**
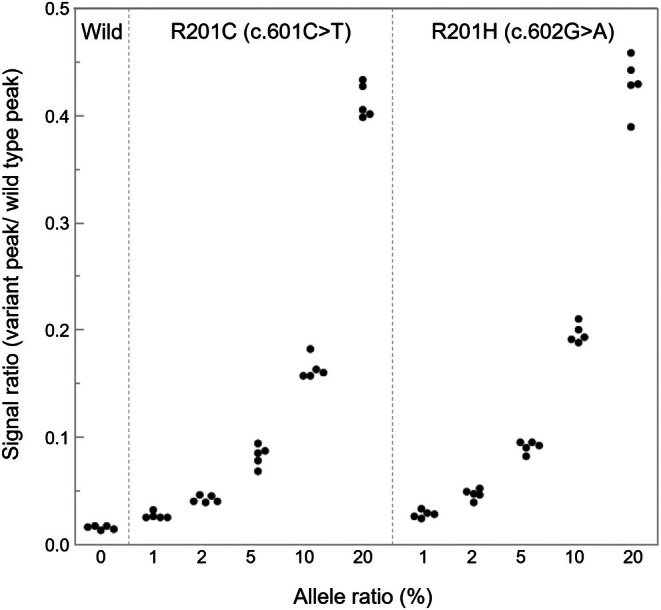
Results of variant detection in mutant sequence plasmids by fragment signal analysis. Both R201C and R201H plasmid samples with mutated allele frequencies of 5% or higher were identified as wild‐type sequence plasmid samples; (R201: 20%: *p* < 0.01, 10%: *p* < 0.01, 5%: *p* = 0.04, 2%: *p* = 0.39, 1%: *p* = 1 / R201H: 20%: *p* < 0.01, 10%: *p* < 0.01, 5%: *p* = 0.04, 2%: *p* = 0.39, 1%: *p* = 1).

The results of variant detection in the mutant sequence plasmids via fragment signal analysis are shown in Figure 4. Both variants (R201C and R201H) were identified in plasmid samples with mutation allele frequencies of 5% or higher, distinguishing them from wild‐type sequence plasmid samples (0% variant allele frequency) (20%, *p* < 0.01; 10%, *p* < 0.01; 5%, *p* = 0.04). However, there was no significant difference at 2% (*p* = 0.39), suggesting that the variant detection sensitivity of this method was between 2% and 5%. The mean SR for variants at 5% allele frequency in plasmid samples was 0.082 for R201C (c.601C>T) and 0.091 for R201H (c.602G>A), indicating that by setting a positive cutoff value of 0.08, it is possible to detect variants in samples with variant allele frequencies of 5% or higher.

#### 3.2.2 Comparison Among Screening Methods

**FIGURE 5:**
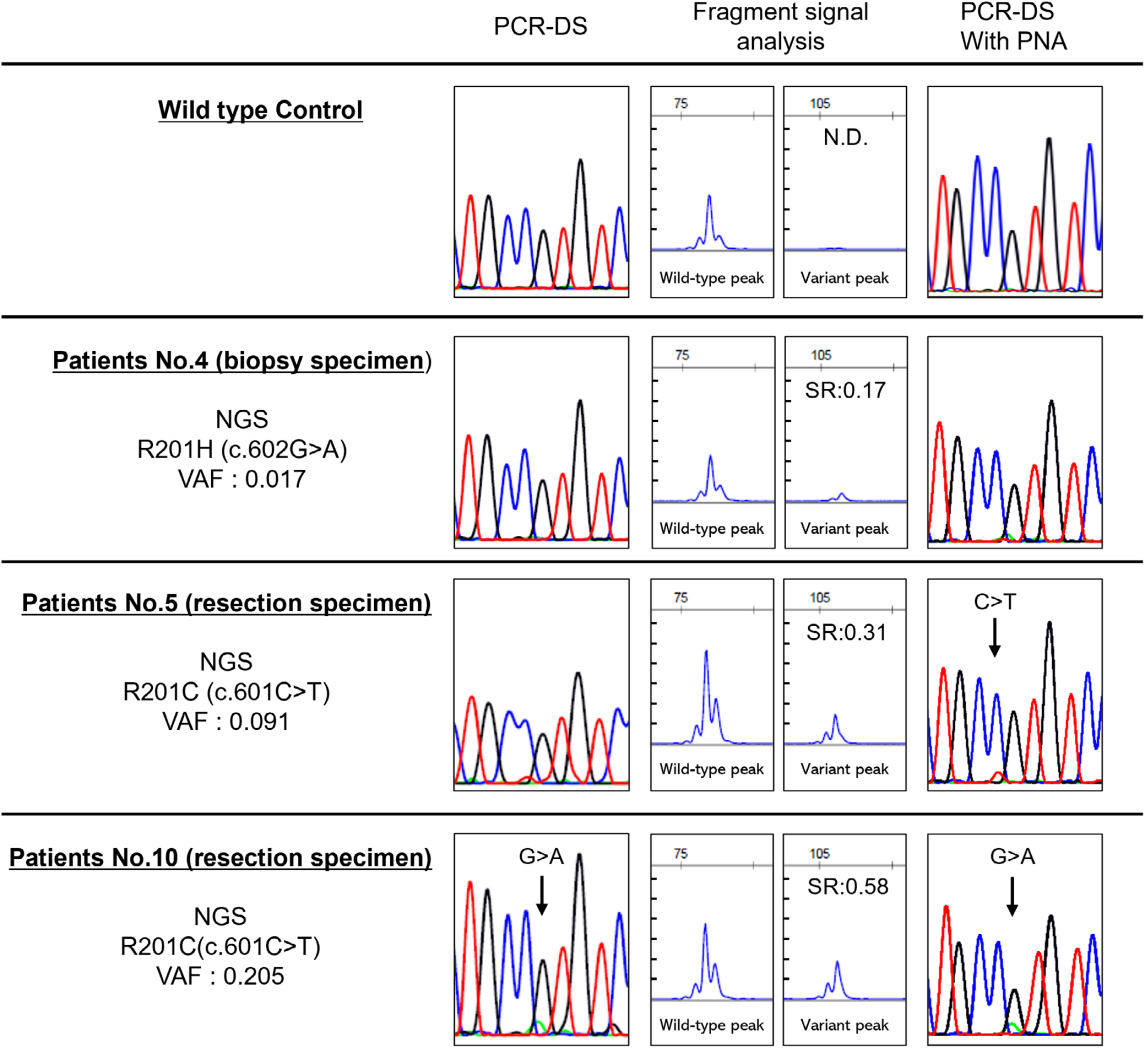
Actual data obtained from the four analysis methods: Wild type, Patient #4 (biopsy specimen), Patient #5 (resection specimen), and Patient #10 (resection specimen). Waveforms identified as variant‐positive by direct sequencing are indicated by arrows.

Table 2 presents the results of these four methods. PCR‐direct sequencing revealed variants in four cases of resected tumor samples (three cases of R201C, one case of R201H) and one biopsy sample (R201C) in IM. Fragment signal analysis revealed positive results in eight cases of resected tumor samples and two biopsy samples in the IM. PCR‐direct sequencing combined with PNA clamping revealed variants in eight resected samples (four cases of R201C and four cases of R201H) and one biopsy sample (R201C) in IM. Representative empirical data for each method are shown in Figure 5. The detection rates of *GNAS*‐positive IM through these three testing measures (PCR‐direct sequencing, fragment signal analysis, PCR‐direct sequencing combined with PNA clamping) were calculated as 0.42, 0.83, and 0.75, respectively, and the detection rate of *GNAS*‐negative LGMFS was calculated to be 1.

**FIGURE 6:**
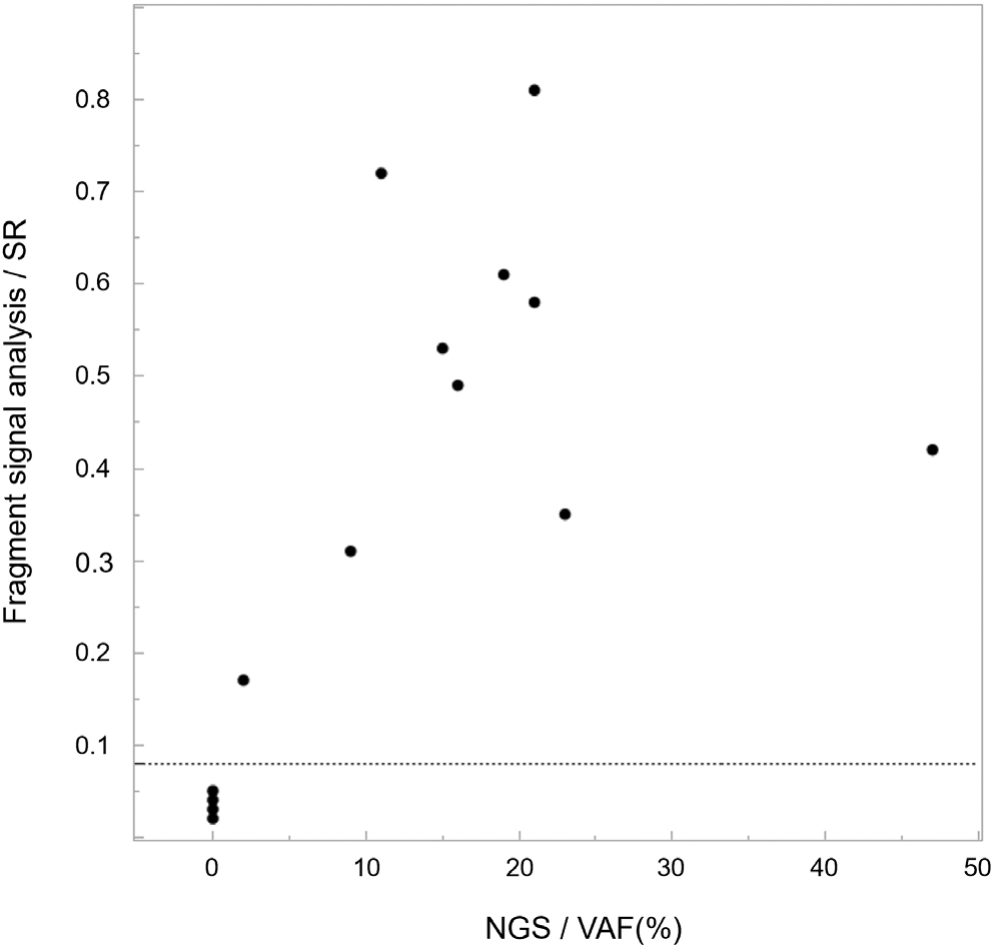
Correlation diagram between the signal ratio (SR) from fragment signal analysis and the variant allele frequency (VAF) from next‐generation sequencing (NGS). The dotted line indicates the SR cutoff value of 0.08.

The SR measured by fragment signal analysis and the VAF measured by NGS showed a correlation coefficient of 0.845 (*p* < 0.01) (Figure 6).

## 4 Discussion

In the current study, a custom Ion Torrent NGS panel‐based analysis for *GNAS* using resected samples detected R201C/R201H variants in 8/10 IM cases and 0/9 LGMFS cases. The R201C/R201H variants were also detected in both available biopsy samples of cases with IM and in neither of the two available biopsy samples of cases with LGMFS. Except for R201C/R201H variants detected only in cases with IM, various variants were detected irrespective of clinical status (IM, LGMFS, normal tissue) or sample conditions (resected, biopsy, relapsed). Sunitsch et al. [18] performed a custom Ion Torrent NGS panel‐based analysis for “hotspot” variants of *GNAS* and other relevant genes in biopsy samples from 13 patients with IM. R201C, R201H, and Q227E were detected in 12/13 cases, and no other missense variants were detected. Libbrecht et al. [21] performed NGS panel‐based analysis using NextSeq on 10 cases of IM. R201C was detected in three cases, and R201H was detected in five cases, with a variant allele frequency of 5%–28%. No variant was detected in one case, and the DNA quality was too low for NGS analysis in one case. This study is the first to demonstrate the molecular characterization of *GNAS* variants of IM through NGS and to confirm that R201C/R201H is the only molecular marker to distinguish IM from LGMFS. Therefore, the detection of R201C/R201H in biopsy samples, if available, would be a reasonable method in a clinical setting, although IM would not be excluded from the absence of the variants.

In the current study, the detection rate of IM codon 201 missense variants using PCR‐direct sequencing was 42%. Willems et al. [16] and Walther et al. [20] reported that the detection rates of PCR‐direct sequencing were 50% and 36.5%, respectively. This low detection rate is potentially due to the extremely small number of tumor cells in the tissue and the possible low occurrence of variant alleles. Delaney et al. [15] proposed co‐amplification at a lower denaturation temperature PCR (COLD‐PCR) as a technique to increase detection sensitivity. COLD‐PCR reduces the proportion of variant allele sequences in the wild‐type allele background by using a low melting temperature resulting from the variant allele and a PCR cycle with a lower denaturation temperature to promote the formation of heteroduplexes [28]. Variants were detected in 61% when combining the use of cases using COLD‐PCR and mutation‐specific restriction enzyme digestion (COLD‐PCR/MSRED) techniques. They reported that the conventional method using PCR and MSRED (PCR/MSRED) was able to detect only 29% of the variants; therefore, this is a useful method to improve diagnostic accuracy.

PNA clamping has been reported to be an effective method for detecting missense variants when the variant allele frequency in tumors is low and the amount of mutated DNA is small [22]. Orum et al. first introduced PNA as a clamp in real‐time PCR to specifically inhibit the amplification of the wild‐type allele and selectively amplify the variant allele with a single base difference [29]. The PNA clamping technique was used to search for *GNAS* codon 201 missense variants. Bianco et al. reported that they were able to detect variants in 100% of fibrous dysplasia tissue samples using direct sequencing combined with PNA clamping [30]. Additionally, Lietman et al. reported that they were able to identify variants in 87.5% of peripheral blood cells associated with fibrous dysplasia and McCune‐Albright syndrome, which are difficult to identify in tissue samples. In the absence of PNA, the detection sensitivity was 25%, which significantly increased the detection sensitivity [25]. However, *GNAS* analysis using PNA clamping for IM has not yet been reported in the literature.

A search for missense variants in *GNAS* codon 201, using restriction enzyme digestion, has also been reported. Candeliere et al. performed nested PCR on fibrous dysplasia tissue, amplified normal DNA that could be digested with restriction enzyme (EagI), and identified variants using gel electrophoresis after restriction enzyme treatment [31]. This method has been used in subsequent studies and has yielded high detection sensitivity when searching for missense variants in *GNAS* codon 201 in fibrous dysplasia [32, 33, 34]. However, because the nucleotide sequence of the restriction enzyme differs by one base from the target codon, two‐step PCR is required to introduce the sequence necessary for restriction enzyme digestion. Therefore, there is a risk of contamination associated with the PCR. The restriction enzyme (BceAI) used in this study has the advantage of requiring only one PCR step because it contains the same base sequence as the target codon. For sequencing, fragment signal analysis was performed using capillary electrophoresis instead of fragment analysis using gel electrophoresis. This method has been used to search for *FLT3* codon 835 missense variants in acute myeloid leukemia and has the advantages of excellent resolution and semi‐quantitative determination [26].

In the current study, we verified whether it is possible to efficiently search for *GNAS* R201 variants with high accuracy using fragment signal analysis with restriction digestion and capillary electrophoresis after PCR combined with PNA clamping, or PCR‐direct sequencing combined with PNA clamping. To the best of our knowledge, there are no reports of the search for *GNAS* missense variants via fragment signal analysis using restriction digestion and capillary electrophoresis after PCR combined with PNA clamping, and this is a new testing method. In resected tumor samples, both methods were able to identify variants in cases where the R201 mutation was confirmed by NGS. Fragment signal analysis of the biopsy samples revealed results similar to those of NGS.

From a clinical perspective, accurate distinction between IM and LGMFS is critical because the two tumors require fundamentally different treatment strategies. IM is a benign lesion for which simple tumor excision is usually sufficient and local recurrence is rare, whereas LGMFS is a malignant tumor that requires wide resection and may necessitate long‐term surveillance and adjuvant therapy. Misclassification based on limited biopsy material can therefore result in either overtreatment of IM or inadequate surgical margins for LGMFS. The ability to detect *GNAS* R201 variants in biopsy specimens provides a valuable adjunct to histopathology and imaging, particularly in diagnostically equivocal cases, and may directly influence surgical margin planning and follow‐up strategies.

**FIGURE 7:**
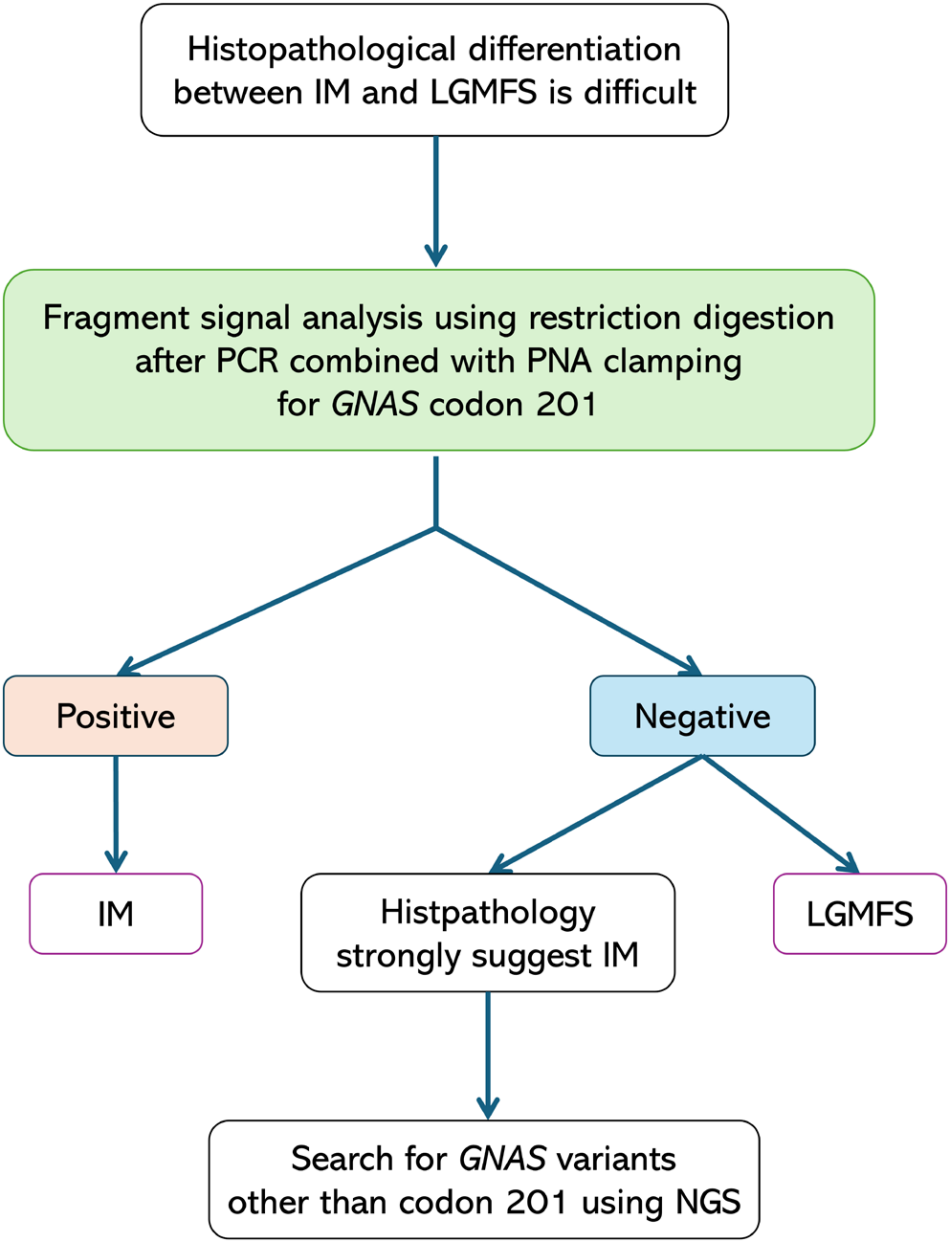
Schematic diagnostic algorithm for differentiating IM from LGMFS using histopathology and stepwise molecular testing.

Based on the results of the current study, we propose the following *GNAS* missense variant search method for IM. In cases where it was difficult to histopathologically differentiate between IM and LGMFS, we first targeted the codon 201 missense variant and performed screening using fragment signal analysis using restriction digestion and capillary electrophoresis after PCR combined with PNA clamping. On the other hand, if the test result is negative but the histopathological findings indicate that IM is more likely, it may be beneficial to perform NGS analysis and determine whether there is a missense variant other than codon 201. The schematic diagnostic algorithm is shown in Figure 7. It should also be noted that variant testing is an auxiliary diagnostic tool, as not all IM cases have *GNAS* variants. It is important to make a final diagnosis in conjunction with histopathological findings.

## 5 Future Directions

Beyond the immediate diagnostic implications for IM and LGMFS, our findings should be considered within the broader context of the evolving role of NGS and biomarker‐driven pathology in soft tissue sarcomas (STS). Recent studies have highlighted how integrated genomic and functional profiling—combining NGS with pharmacologic testing or three‐dimensional culture assays—can refine sarcoma classification, identify biologically distinct subgroups, and uncover actionable therapeutic vulnerabilities, including in MFS [35, 36, 37]. These approaches underscore a paradigm shift in STS pathology, in which molecular biomarkers increasingly complement conventional histopathological evaluation.

While comprehensive genomic and functional profiling strategies offer deep biological insight, their complexity and resource requirements may limit widespread implementation in routine diagnostic practice. In contrast, the present study demonstrates how a focused, disease‐defining hotspot variant can be leveraged as a practical and clinically actionable diagnostic biomarker. By systematically comparing multiple detection methodologies and translating NGS‐based molecular knowledge into a simplified screening workflow, we bridge advanced genomic pathology with real‐world clinical applicability.

From a broader perspective, our results support a stepwise, biomarker‐driven diagnostic framework for STS. In this model, targeted molecular assays—such as *GNAS* R201 variant testing—serve as first‐line tools to support histopathological interpretation in diagnostically challenging cases, while more comprehensive genomic or pharmacologic profiling may be reserved for selected cases requiring further biological or therapeutic stratification. Such an approach maximizes diagnostic accuracy while maintaining feasibility and cost‐effectiveness in daily practice.

Finally, *GNAS* codon 201 variants are not unique to IM and have been identified in a range of benign and neoplastic conditions, including fibrous dysplasia [38], intraductal papillary mucinous neoplasms [39], low‐grade appendiceal mucinous neoplasms [40], colorectal villous adenomas [41], and McCune–Albright syndrome [25]. Given that some of these entities pose diagnostic challenges or carry a risk of malignant transformation, future studies evaluating the applicability of our targeted variant detection strategy in these settings are warranted.

## 6 Conclusion

A custom NGS panel‐based analysis for *GNAS* revealed molecular characterization of IM and LGMFS, representing R201C/R201H as diagnostic evidence for IM and demonstrating various sequence alterations irrespective of clinical status (IM, LGMFS, and normal) or sample conditions (resected, biopsy, and relapsed). Fragment signal analysis using restriction digestion and capillary electrophoresis after PCR combined with PNA clamping, preferably using biopsy samples, could be a reasonable diagnostic method for distinguishing IM from LGMFS in clinical settings. Furthermore, negative results for *GNAS* R201C/R201H variants did not exclude the diagnosis of IM.

Within the broader framework of biomarker‐driven pathology in soft tissue sarcomas, our findings illustrate how targeted, disease‐specific molecular assays can effectively complement histopathological evaluation and more comprehensive genomic profiling in routine clinical practice.

## Author Contributions

**Munehisa Kito:** conceptualization, methodology, validation, formal analysis, writing – original draft, funding acquisition, visualization. **Kaoru Aoki:** investigation, resources. **Shohei Shigeto:** conceptualization, methodology, validation, formal analysis, writing – original draft. **Hirokazu Ideta:** investigation, resources. **Atsushi Tanaka:** investigation, resources. **Kenji Sano:** investigation, resources. **Akira Takazawa:** investigation, resources. **Tomoki Kosho:** methodology, writing – review and editing. **Mai Iwaya:** investigation, resources. **Tomomi Yamaguchi:** validation, investigation, resources. **Masanori Okamoto:** investigation, resources, writing – review and editing, supervision. **Hiromasa Hasegawa:** investigation, writing – review and editing. **Jun Takahashi:** writing – review and editing. **Tomomi Fujikawa:** investigation, resources.

## Funding

This work was supported by the Japan Society for the Promotion of Science (Grant 21K16650).

## Ethics Statement

This study design was approved by the institutional ethics review board of Shinshu University School of Medicine (Approval Number: 688).

## Conflicts of Interest

The authors declare no conflicts of interest.

## Supporting information


**Table S1.** Comprehensive variant characterization of *GNAS*.

**Data S1:** Supporting Information.

## Data Availability

The datasets used and/or analyzed for the current study are available from the corresponding author upon reasonable request.

## References

[1] F. M. Enzinger , “Intramuscular Myxoma; a Review and Follow‐Up Study of 34 Cases,” American Journal of Clinical Pathology 43 (1965): 104–113. 14253111 10.1093/ajcp/43.2.104

[2] G. P. Nielsen , J. X. O'Connell , and A. E. Rosenberg , “Intramuscular Myxoma: A Clinicopathologic Study of 51 Cases With Emphasis on Hypercellular and Hypervascular Variants,” American Journal of Surgical Pathology 22, no. 10 (1998): 1222–1227. 9777984 10.1097/00000478-199810000-00007

[3] J. F. van Roggen , M. E. McMenamin , and C. D. Fletcher , “Cellular Myxoma of Soft Tissue: A Clinicopathological Study of 38 Cases Confirming Indolent Clinical Behaviour,” Histopathology 39, no. 3 (2001): 287–297. 11532040 10.1046/j.1365-2559.2001.01209.x

[4] H. S. Schwartz and R. Walker , “Recognizable Magnetic Resonance Imaging Characteristics of Intramuscular Myxoma,” Orthopedics 20, no. 5 (1997): 431–435. 9172250 10.3928/0147-7447-19970501-12

[5] M. D. Murphey , G. A. McRae , J. C. Fanburg‐Smith , H. T. Temple , A. M. Levine , and A. J. Aboulafia , “Imaging of Soft‐Tissue Myxoma With Emphasis on CT and MR and Comparison of Radiologic and Pathologic Findings,” Radiology 225, no. 1 (2002): 215–224. 12355008 10.1148/radiol.2251011627

[6] A. Luna , S. Martinez , and E. Bossen , “Magnetic Resonance Imaging of Intramuscular Myxoma With Histological Comparison and a Review of the Literature,” Skeletal Radiology 34, no. 1 (2005): 19–28. 15538560 10.1007/s00256-004-0848-9

[7] S. Sukpanichyingyong , S. Matsumoto , K. Ae , et al., “Surgical Treatment of Intramuscular Myxoma,” Indian Journal of Orthopaedics 55, no. 4 (2021): 892–897. 34194644 10.1007/s43465-021-00367-9 PMC8192669

[8] A. Reiter , K. Trumm , T. M. Ballhause , et al., “Diagnostic and Therapeutic Pathways of Intramuscular Myxoma,” Diagnostics (Basel) 12, no. 7 (2022): 1573. 35885479 10.3390/diagnostics12071573 PMC9316518

[9] J. F. van Graadt Roggen , P. C. Hogendoorn , and C. D. Fletcher , “Myxoid Tumours of Soft Tissue,” Histopathology 35, no. 4 (1999): 291–312. 10564384 10.1046/j.1365-2559.1999.00835.x

[10] L. A. Doyle , E. Möller , P. Dal Cin , C. D. Fletcher , F. Mertens , and J. L. Hornick , “MUC4 Is a Highly Sensitive and Specific Marker for Low‐Grade Fibromyxoid Sarcoma,” American Journal of Surgical Pathology 35, no. 5 (2011): 733–741. 21415703 10.1097/PAS.0b013e318210c268

[11] T. Mentzel , E. Calonje , C. Wadden , et al., “Myxofibrosarcoma. Clinicopathologic Analysis of 75 Cases With Emphasis on the Low‐Grade Variant,” American Journal of Surgical Pathology 20, no. 4 (1996): 391–405. 8604805 10.1097/00000478-199604000-00001

[12] H. Y. Huang , P. Lal , J. Qin , M. F. Brennan , and C. R. Antonescu , “Low‐Grade Myxofibrosarcoma: A Clinicopathologic Analysis of 49 Cases Treated at a Single Institution With Simultaneous Assessment of the Efficacy of 3‐Tier and 4‐Tier Grading Systems,” Human Pathology 35, no. 5 (2004): 612–621. 15138937 10.1016/j.humpath.2004.01.016

[13] T. Fujiwara , Y. Kaneuchi , Y. Tsuda , J. Stevenson , M. Parry , and L. Jeys , “Low‐Grade Soft‐Tissue Sarcomas: What Is an Adequate Margin for Local Disease Control?,” Surgical Oncology 35 (2020): 303–308. 32961431 10.1016/j.suronc.2020.08.022

[14] S. Okamoto , M. Hisaoka , M. Ushijima , S. Nakahara , S. Toyoshima , and H. Hashimoto , “Activating Gs(Alpha) Mutation in Intramuscular Myxomas With and Without Fibrous Dysplasia of Bone,” Virchows Archiv 437, no. 2 (2000): 133–137. 10993273 10.1007/s004280000217

[15] D. Delaney , T. C. Diss , N. Presneau , et al., “GNAS1 Mutations Occur More Commonly Than Previously Thought in Intramuscular Myxoma,” Modern Pathology 22, no. 5 (2009): 718–724. 19287459 10.1038/modpathol.2009.32

[16] S. M. Willems , A. B. Mohseny , C. Balog , et al., “Cellular/Intramuscular Myxoma and Grade I Myxofibrosarcoma Are Characterized by Distinct Genetic Alterations and Specific Composition of Their Extracellular Matrix,” Journal of Cellular and Molecular Medicine 13, no. 7 (2009): 1291–1301. 19320777 10.1111/j.1582-4934.2009.00747.x PMC4496143

[17] T. Kimura , S. P. Pydi , J. Pham , and N. Tanaka , “Metabolic Functions of G Protein‐Coupled Receptors in Hepatocytes‐Potential Applications for Diabetes and NAFLD,” Biomolecules 10, no. 10 (2020): 1445. 33076386 10.3390/biom10101445 PMC7602561

[18] S. Sunitsch , M. M. Gilg , K. Kashofer , F. Gollowitsch , A. Leithner , and B. Liegl‐Atzwanger , “Detection of GNAS Mutations in Intramuscular / Cellular Myxomas as Diagnostic Tool in the Classification of Myxoid Soft Tissue Tumors,” Diagnostic Pathology 13, no. 1 (2018): 52. 30111377 10.1186/s13000-018-0734-8 PMC6094570

[19] E. M. Bekers , A. Eijkelenboom , P. Rombout , et al., “Identification of Novel GNAS Mutations in Intramuscular Myxoma Using Next‐Generation Sequencing With Single‐Molecule Tagged Molecular Inversion Probes,” Diagnostic Pathology 14, no. 1 (2019): 15. 30736805 10.1186/s13000-019-0787-3 PMC6368757

[20] I. Walther , B. M. Walther , Y. Chen , and I. Petersen , “Analysis of GNAS1 Mutations in Myxoid Soft Tissue and Bone Tumors,” Pathology, Research and Practice 210, no. 1 (2014): 1–4. 24268734 10.1016/j.prp.2013.09.003

[21] L. Libbrecht , I. V. Bempt , T. Schubert , R. Sciot , and C. Galant , “Next Generation Sequencing for GNAS Uncovers CD34 as a Sensitive Marker for Intramuscular Myxoma,” Annals of Diagnostic Pathology 43 (2019): 151409. 31726379 10.1016/j.anndiagpath.2019.151409

[22] M. F. Fouz and D. H. Appella , “PNA Clamping in Nucleic Acid Amplification Protocols to Detect Single Nucleotide Mutations Related to Cancer,” Molecules 25, no. 4 (2020): 786. 32059456 10.3390/molecules25040786 PMC7070360

[23] C. C. Chiou , J. D. Luo , and T. L. Chen , “Single‐Tube Reaction Using Peptide Nucleic Acid as Both PCR Clamp and Sensor Probe for the Detection of Rare Mutations,” Nature Protocols 1, no. 6 (2006): 2604–2612. 17406515 10.1038/nprot.2006.428

[24] J. D. Luo , E. C. Chan , C. L. Shih , et al., “Detection of Rare Mutant K‐Ras DNA in a Single‐Tube Reaction Using Peptide Nucleic Acid as Both PCR Clamp and Sensor Probe,” Nucleic Acids Research 34, no. 2 (2006): e12. 16432256 10.1093/nar/gnj008 PMC1345699

[25] S. A. Lietman , C. Ding , and M. A. Levine , “A Highly Sensitive Polymerase Chain Reaction Method Detects Activating Mutations of the GNAS Gene in Peripheral Blood Cells in McCune‐Albright Syndrome or Isolated Fibrous Dysplasia,” Journal of Bone and Joint Surgery. American Volume 87, no. 11 (2005): 2489–2494. 16264125 10.2106/JBJS.E.00160

[26] K. M. Murphy , M. Levis , M. J. Hafez , et al., “Detection of FLT3 Internal Tandem Duplication and D835 Mutations by a Multiplex Polymerase Chain Reaction and Capillary Electrophoresis Assay,” Journal of Molecular Diagnostics 5, no. 2 (2003): 96–102. 10.1016/S1525-1578(10)60458-8 PMC1907323 12707374

[27] WHO Classification of Tumours Editorial Board , ed., World Health Organization Classification of Soft Tissue and Bone Tumours, 5th ed. (IARC Press, 2020).

[28] J. Li , L. Wang , H. Mamon , M. H. Kulke , R. Berbeco , and G. M. Makrigiorgos , “Replacing PCR With COLD‐PCR Enriches Variant DNA Sequences and Redefines the Sensitivity of Genetic Testing,” Nature Medicine 14, no. 5 (2008): 579–584. 10.1038/nm1708 18408729

[29] H. Orum , P. E. Nielsen , M. Egholm , R. H. Berg , O. Buchardt , and C. Stanley , “Single Base Pair Mutation Analysis by PNA Directed PCR Clamping,” Nucleic Acids Research 21, no. 23 (1993): 5332–5336. 8265345 10.1093/nar/21.23.5332 PMC310567

[30] P. Bianco , M. Riminucci , A. Majolagbe , et al., “Mutations of the GNAS1 Gene, Stromal Cell Dysfunction, and Osteomalacic Changes in Non‐McCune‐Albright Fibrous Dysplasia of Bone,” Journal of Bone and Mineral Research 15, no. 1 (2000): 120–128. 10646121 10.1359/jbmr.2000.15.1.120

[31] G. A. Candeliere , P. J. Roughley , and F. H. Glorieux , “Polymerase Chain Reaction‐Based Technique for the Selective Enrichment and Analysis of Mosaic arg201 Mutations in G Alpha s From Patients With Fibrous Dysplasia of Bone,” Bone 21, no. 2 (1997): 201–206. 9267696 10.1016/s8756-3282(97)00107-5

[32] A. Sakamoto , Y. Oda , Y. Iwamoto , and M. Tsuneyoshi , “A Comparative Study of Fibrous Dysplasia and Osteofibrous Dysplasia With Regard to Gsalpha Mutation at the Arg201 Codon: Polymerase Chain Reaction‐Restriction Fragment Length Polymorphism Analysis of Paraffin‐Embedded Tissues,” Journal of Molecular Diagnostics 2, no. 2 (2000): 67–72. 10.1016/s1525-1578(10)60618-6 PMC1906902 11272890

[33] N. Kalfa , P. Philibert , F. Audran , et al., “Searching for Somatic Mutations in McCune‐Albright Syndrome: A Comparative Study of the Peptidic Nucleic Acid Versus the Nested PCR Method Based on 148 DNA Samples,” European Journal of Endocrinology 155, no. 6 (2006): 839–843. 17132753 10.1530/eje.1.02301

[34] S. Toyosawa , M. Yuki , M. Kishino , et al., “Ossifying Fibroma vs Fibrous Dysplasia of the Jaw: Molecular and Immunological Characterization,” Modern Pathology 20, no. 3 (2007): 389–396. 17334331 10.1038/modpathol.3800753

[35] S. Vanni , V. Fausti , E. Fonzi , et al., “Unveiling the Genomic Basis of Chemosensitivity in Sarcomas of the Extremities: An Integrated Approach for an Unmet Clinical Need,” International Journal of Molecular Sciences 24, no. 8 (2023): 6926. 37108089 10.3390/ijms24086926 PMC10138892

[36] F. de Nigris , C. Meo , and W. Palinski , “Combination of Genomic Landsscape and 3D Culture Functional Assays Bridges Sarcoma Phenotype to Target and Immunotherapy,” Cells 12, no. 17 (2023): 2204. 37681936 10.3390/cells12172204 PMC10486752

[37] C. Caruso and C. Garofalo , “Pharmacogenomics Biomarkers of Soft Tissue Sarcoma Therapies,” Frontiers in Oncology 10 (2020): 509. 32351891 10.3389/fonc.2020.00509 PMC7174622

[38] S. E. Lee , E. H. Lee , H. Park , et al., “The Diagnostic Utility of the GNAS Mutation in Patients With Fibrous Dysplasia: Meta‐Analysis of 168 Sporadic Cases,” Human Pathology 43, no. 8 (2012): 1234–1242. 22245114 10.1016/j.humpath.2011.09.012

[39] M. C. Tan , O. Basturk , A. R. Brannon , et al., “GNAS and KRAS Mutations Define Separate Progression Pathways in Intraductal Papillary Mucinous Neoplasm‐Associated Carcinoma,” Journal of the American College of Surgeons 220, no. 5 (2015): 845–854.e1. 25840541 10.1016/j.jamcollsurg.2014.11.029 PMC4409519

[40] G. Nishikawa , S. Sekine , R. Ogawa , et al., “Frequent GNAS Mutations in Low‐Grade Appendiceal Mucinous Neoplasms,” British Journal of Cancer 108, no. 4 (2013): 951–958. 23403822 10.1038/bjc.2013.47 PMC3590682

[41] M. Yamada , S. Sekine , R. Ogawa , et al., “Frequent Activating GNAS Mutations in Villous Adenoma of the Colorectum,” Journal of Pathology 228, no. 1 (2012): 113–118. 22374786 10.1002/path.4012

