# Supplementary material for: Molecular Characterization and Its Clinical Application of GNAS Variants in Intramuscular Myxoma
Source: Cancer Med. 2026 Mar 29;15(4):e71751. doi: 10.1002/cam4.71751 (PMC13140542; doi:10.1002/cam4.71751)
Supplement: Supplementary file 1 — Table S1. Comprehensive variant characterization of GNAS. [file CAM4-15-e71751-s001.docx]

Supplemental Table S1. Comprehensive variant characterization of *GNAS*

| No. | diagnosis | specimen | VAF | coverage of depth | variant type | variant (NM_000516.5) | chr | pos (hg19) | ref | alt | ada_score | rf_score | SIFT | LRT | Mutation  Taster | Mutation  Assessor | FATHMM | PROVEAN | MetaSVM | MetaLR | M-CAP | fathmm-MKL | PCR-DS | Fragment signal analysis (SR) | PCR-DS with PNA |
| --- | --- | --- | --- | --- | --- | --- | --- | --- | --- | --- | --- | --- | --- | --- | --- | --- | --- | --- | --- | --- | --- | --- | --- | --- | --- |
| 1 | IM | Resection | 0.032 | 2629 | missense variant | c.75G>T,p.Lys25Asn | 20 | 57466856 | G | T |  |  | T | - | D | L | D | N | T | T | D | D |  |  |  |
|  |  |  | 0.036 | 1854 | missense variant | c.181A>G,p.Arg61Gly | 20 | 57470708 | A | G |  |  | D | D | D | H | D | D | D | D | D | D |  |  |  |
|  |  |  | 0.067 | 1005 | synonymous variant | c.231G>T,p.Pro77= | 20 | 57474014 | G | T |  |  | - | - | - | - | - | - | - | - | - | - |  |  |  |
|  |  |  | 0.061 | 1005 | missense variant | c.248A>G,p.Asn83Ser | 20 | 57474031 | A | G |  |  | D | N | D | L | D | N | T | T | D | D |  |  |  |
|  |  |  | 0.020 | 1004 | splice site variant | c.257+3A>T | 20 | 57474043 | A | T | 0.99986098 | 0.956 | - | - | - | - | - | - | - | - | - | - |  |  |  |
|  |  |  | 0.024 | 941 | synonymous variant | c.573G>A,p.Val191= | 20 | 57484259 | G | A |  |  | - | - | - | - | - | - | - | - | - | - |  |  |  |
|  |  |  | 0.000 | 1114 |  | (c.601C>T,p.Arg201Cys) |  |  |  |  |  |  |  |  |  |  |  |  |  |  |  |  | - | + (0.42) | - |
|  |  |  | 0.473 | 1114 | missense variant | c.602G>A,p.Arg201His | 20 | 57484421 | G | A |  |  | D | D | D | H | D | D | D | D | D | D | - |  | R201H |
|  |  |  | 0.033 | 1101 | missense variant | c.676G>A,p.Gly226Ser | 20 | 57484592 | G | A |  |  | D | D | D | H | D | D | D | D | D | D |  |  |  |
|  |  |  | 0.059 | 1101 | missense variant | c.695G>T,p.Arg232Leu | 20 | 57484611 | G | T |  |  | D | D | D | H | D | D | D | D | D | D |  |  |  |
|  |  |  | 0.120 | 815 | missense variant | c.797T>C,p.Leu266Pro | 20 | 57484817 | T | C |  |  | D | D | D | H | D | D | D | D | D | D |  |  |  |
|  |  |  | 0.032 | 1356 | missense variant | c.822G>T,p.Lys274Asn | 20 | 57484842 | G | T |  |  | D | D | D | M | D | D | D | D | D | D |  |  |  |
|  |  |  | 0.028 | 1244 | missense variant | c.1001T>C,p.Val334Ala | 20 | 57485419 | T | C |  |  | T | D | D | M | D | D | D | D | D | D |  |  |  |
|  |  |  | 0.111 | 768 | missense variant | c.1066C>T,p.Arg356Cys | 20 | 57485765 | C | T |  |  | D | D | D | H | D | D | D | D | D | D |  |  |  |
| 2 | IM | Biopsy | 0.164 | 10367 | missense variant | c.601C>T,p.Arg201Cys | 20 | 57484420 | C | T |  |  | D | D | D | H | D | D | D | D | D | D | R201C | + (0.49) | R201C |
|  |  |  | 0.000 | 10364 |  | (c.602G>A,p.Arg201His) |  |  |  |  |  |  |  |  |  |  |  |  |  |  |  |  | - |  | - |
|  |  | Resection | 0.210 | 9117 | missense variant | c.601C>T,p.Arg201Cys | 20 | 57484420 | C | T |  |  | D | D | D | H | D | D | D | D | D | D | R201C | + (0.81) | R201C |
|  |  |  | 0.000 | 9116 |  | (c.602G>A,p.Arg201His) |  |  |  |  |  |  |  |  |  |  |  |  |  |  |  |  | - |  | - |
| 3 | IM | Resection | 0.000 | 9567 |  | (c.601C>T,p.Arg201Cys) |  |  |  |  |  |  |  |  |  |  |  |  |  |  |  |  | - | - (0.04) | - |
|  |  |  | 0.000 | 9569 |  | (c.602G>A,p.Arg201His) |  |  |  |  |  |  |  |  |  |  |  |  |  |  |  |  | - |  | - |
| 4 | IM | Biopsy | 0.000 | 10929 |  | (c.601C>T,p.Arg201Cys) |  |  |  |  |  |  |  |  |  |  |  |  |  |  |  |  | - | + (0.17) | - |
|  |  |  | 0.017 | 10926 | missense variant | c.602G>A,p.Arg201His | 20 | 57484421 | G | A |  |  | D | D | D | H | D | D | D | D | D | D | - |  | - |
|  |  |  | 0.027 | 11192 | missense variant | c.847C>T,p.Arg283Cys | 20 | 57485013 | C | T |  |  | T | D | D | M | D | D | D | D | D | D |  |  |  |
|  |  | Resection | 0.000 | 13135 |  | (c.601C>T,p.Arg201Cys) |  |  |  |  |  |  | D | D | D | H | D | D | D | D | D | D | - | + (0.61) | - |
|  |  |  | 0.187 | 13131 | missense variant | c.602G>A,p.Arg201His | 20 | 57484421 | G | A |  |  | D | D | D | H | D | D | D | D | D | D | - |  | R201H |
| 5 | IM | Resection | 0.034 | 5505 | missense variant | c.217G>A,p.Gly73Ser | 20 | 57474000 | G | A |  |  | T | U | D | N | D | N | D | D | D | D |  |  |  |
|  |  |  | 0.019 | 5994 | synonymous variant | c.348C>T,p.Pro116= | 20 | 57478762 | C | T |  |  | - | - | - | - | - | - | - | - | - | - |  |  |  |
|  |  |  | 0.091 | 5155 | missense variant | c.601C>T,p.Arg201Cys | 20 | 57484420 | C | T |  |  | D | D | D | H | D | D | D | D | D | D | - | + (0.31) | R201C |
|  |  |  | 0.000 | 5156 |  | (c.602G>A,p.Arg201His) |  |  |  |  |  |  |  |  |  |  |  |  |  |  |  |  | - |  | - |
| 6 | IM | Resection | 0.028 | 4024 | synonymous variant | c.105G>A,p.Gln35= | 20 | 57466886 | G | A |  |  | - | - | - | - | - | - | - | - | - | - |  |  |  |
|  |  |  | 0.227 | 590 | missense variant | c.601C>T,p.Arg201Cys | 20 | 57484420 | C | T |  |  | D | D | D | H | D | D | D | D | D | D | R201C | + (0.35) | R201C |
|  |  |  | 0.000 | 590 |  | (c.602G>A,p.Arg201His) |  |  |  |  |  |  |  |  |  |  |  |  |  |  |  |  | - |  | - |
|  |  |  | 0.026 | 589 | missense variant | c.628A>C,p.Thr210Pro | 20 | 57484447 | A | C |  |  | D | D | D | H | D | D | D | D | D | D |  |  |  |
|  |  |  | 0.023 | 2175 | missense variant | c.740T>C,p.Val247Ala | 20 | 57484760 | T | C |  |  | D | D | D | H | D | D | D | D | D | D |  |  |  |
|  |  |  | 0.118 | 1343 | missense variant | c.949C>T,p.Arg317Cys | 20 | 57485115 | C | T |  |  | D | U | D | M | D | D | D | D | D | D |  |  |  |
|  |  |  | 0.094 | 1291 | missense variant | c.1000G>C,p.Val334Leu | 20 | 57485418 | G | C |  |  | T | D | D | M | D | D | D | D | D | D |  |  |  |
|  |  |  | 0.026 | 1290 | missense variant | c.1010C>T,p.Ala337Val | 20 | 57485428 | C | T |  |  | D | D | D | M | D | D | D | D | D | D |  |  |  |
|  |  |  | 0.037 | 351 | missense variant | c.1055G>A,p.Ser352Asn | 20 | 57485754 | G | A |  |  | T | D | D | L | D | N | D | D | D | D |  |  |  |
|  |  | Normal | 0.241 | 372 | intron_variant | c.140-10C>T | 20 | 57470657 | C | T | 0.03630626 | 0.366 | - | - | - | - | - | - | - | - | - | - |  |  |  |
|  |  |  | 0.067 | 862 | missense variant | c.220G>A,p.Glu74Lys | 20 | 57474003 | G | A |  |  | D | U | D | N | D | N | T | T | D | D |  |  |  |
|  |  |  | 0.038 | 865 | synonymous variant | c.228C>T,p.Asp76= | 20 | 57474011 | C | T |  |  | - | - | - | - | - | - | - | - | - | - |  |  |  |
|  |  |  | 0.065 | 863 | missense variant (splice site variant) | c.256G>A,p.Gly86Ser | 20 | 57474039 | G | A | 0.96526675 | 0.678 | T | N | D | N | D | N | T | T | D | D |  |  |  |
|  |  |  | 0.023 | 861 | missense variant | c.308T>C,p.Ile103Thr | 20 | 57478636 | T | C |  |  | . | D | D | M | . | . | D | D | D | D |  |  |  |
|  |  |  | 0.127 | 861 | splice site variant | c.312+4C>T | 20 | 57478644 | C | T | 2.7719E-05 | 0.004 | - | - | - | - | - | - | - | - | - | - |  |  |  |
|  |  |  | 0.000 | 486 |  | (c.601C>T,p.Arg201Cys) |  |  |  |  |  |  |  |  |  |  |  |  |  |  |  |  |  |  |  |
|  |  |  | 0.000 | 486 |  | (c.602G>A,p.Arg201His) |  |  |  |  |  |  |  |  |  |  |  |  |  |  |  |  |  |  |  |
|  |  |  | 0.049 | 1565 | nonsense variant | c.679C>T,p.Gln227* | 20 | 57484595 | C | T |  |  | - | D | A | - | - | - | - | - | - | D |  |  |  |
|  |  |  | 0.089 | 1323 | missense variant | c.775G>A,p.Glu259Lys | 20 | 57484795 | G | A |  |  | D | D | D | H | D | D | D | D | D | D |  |  |  |
|  |  |  | 0.043 | 2417 | missense variant | c.1004C>T,p.Thr335Ile | 20 | 57485422 | C | T |  |  | T | N | D | L | D | D | D | D | D | D |  |  |  |
|  |  |  | 0.025 | 2418 | missense variant | c.1018T>C,p.Phe340Leu | 20 | 57485436 | T | C |  |  | D | D | D | M | D | D | D | D | D | D |  |  |  |
|  |  |  | 0.024 | 2418 | synonymous variant | c.1023T>C,p.Ile341= | 20 | 57485441 | T | C |  |  | - | - | - | - | - | - | - | - | - | - |  |  |  |
|  |  |  | 0.075 | 1048 | splice site variant | c.1039-5C>T | 20 | 57485733 | C | T | 0.00013571 | 0.02 | - | - | - | - | - | - | - | - | - | - |  |  |  |
|  |  |  | 0.024 | 1061 | missense variant | c.1096G>A,p.Ala366Thr | 20 | 57485795 | G | A |  |  | D | D | D | H | D | D | D | D | D | D |  |  |  |
| 7 | IM | Resection | 0.000 | 12404 |  | (c.601C>T,p.Arg201Cys) |  |  |  |  |  |  |  |  |  |  |  |  |  |  |  |  | - | - (0.05) | - |
|  |  |  | 0.000 | 12404 |  | (c.602G>A,p.Arg201His) |  |  |  |  |  |  |  |  |  |  |  |  |  |  |  |  | - |  | - |
| 8 | IM | Resection | 0.000 | 11508 |  | (c.601C>T,p.Arg201Cys) |  |  |  |  |  |  |  |  |  |  |  |  |  |  |  |  | - | + (0.53) | - |
|  |  |  | 0.153 | 11503 | missense variant | c.602G>A,p.Arg201His | 20 | 57484421 | G | A |  |  | D | D | D | H | D | D | D | D | D | D | - |  | R201H |
| 9 | IM | Resection | 0.114 | 14812 | missense variant | c.601C>T,p.Arg201Cys | 20 | 57484420 | C | T |  |  | D | D | D | H | D | D | D | D | D | D | R201C | + (0.72) | R201C |
|  |  |  | 0.000 | 14808 |  | (c.602G>A,p.Arg201His) |  |  |  |  |  |  |  |  |  |  |  |  |  |  |  |  | - |  | - |
| 10 | IM | Resection | 0.025 | 4602 | splice site variant | c.258-7A>G | 20 | 57478579 | A | G |  |  | - | - | - | - | - | - | - | - | - | - |  |  |  |
|  |  |  | 0.000 | 5355 |  | (c.601C>T,p.Arg201Cys) |  |  |  |  |  |  |  |  |  |  |  |  |  |  |  |  | - | + (0.58) | - |
|  |  |  | 0.205 | 5350 | missense variant | c.602G>A,p.Arg201His | 20 | 57484421 | G | A |  |  | D | D | D | H | D | D | D | D | D | D | R201H |  | R201H |
| 11 | LGMFS | Resection | - | 0 |  | (c.601C>T,p.Arg201Cys) |  |  |  |  |  |  |  |  |  |  |  |  |  |  |  |  | - | - (0.02) | - |
|  |  |  | - | 0 |  | (c.602G>A,p.Arg201His) |  |  |  |  |  |  |  |  |  |  |  |  |  |  |  |  | - |  | - |
|  |  |  | 0.025 | 242 | synonymous variant | c.939A>G,p.Pro313= | 20 | 57485105 | A | G |  |  | - | - | - | - | - | - | - | - | - | - |  |  |  |
| 12 | LGMFS | Resection | 0.000 | 14628 |  | (c.601C>T,p.Arg201Cys) |  |  |  |  |  |  |  |  |  |  |  |  |  |  |  |  | - | - (0.03) | - |
|  |  |  | 0.000 | 14622 |  | (c.602G>A,p.Arg201His) |  |  |  |  |  |  |  |  |  |  |  |  |  |  |  |  | - |  | - |
| 13 | LGMFS | Resection | 0.034 | 4402 | synonymous variant | c.183G>A,p.Arg61= | 20 | 57470710 | G | A |  |  | - | - | - | - | - | - | - | - | - | - |  |  |  |
|  |  |  | 0.043 | 3087 | synonymous variant | c.231G>A,p.Pro77= | 20 | 57474014 | G | A |  |  | - | - | - | - | - | - | - | - | - | - |  |  |  |
|  |  |  | 0.037 | 3089 | missense variant | c.253G>A,p.Asp85Asn | 20 | 57474036 | G | A |  |  | D | N | D | N | D | N | D | D | D | D |  |  |  |
|  |  |  | 0.021 | 1752 | synonymous variant | c.300A>G,p.Lys100= | 20 | 57478628 | A | G |  |  | - | - | - | - | - | - | - | - | - | - |  |  |  |
|  |  |  | 0.025 | 3180 | splice site variant | c.432+1G>T | 20 | 57478847 | G | T | 0.99998553 | 0.936 | - | - | - | - | - | - | - | - | - | - |  |  |  |
|  |  |  | 0.039 | 6173 | missense variant | c.479G>A,p.Arg160His | 20 | 57480484 | G | A |  |  | - | N | D | L | D | - | T | T | D | D |  |  |  |
|  |  |  | 0.027 | 6179 | missense variant | c.502G>A,p.Glu168Lys | 20 | 57480507 | G | A |  |  | D | D | D | M | D | D | D | D | D | D |  |  |  |
|  |  |  | 0.028 | 3853 | synonymous variant | c.579C>T,p.Ser193= | 20 | 57484265 | C | T |  |  | - | - | - | - | - | - | - | - | - | - |  |  |  |
|  |  |  | 0.000 | 2138 |  | (c.601C>T,p.Arg201Cys) |  |  |  |  |  |  |  |  |  |  |  |  |  |  |  |  | - | - (0.03) | - |
|  |  |  | 0.000 | 2137 |  | (c.602G>A,p.Arg201His) |  |  |  |  |  |  |  |  |  |  |  |  |  |  |  |  | - |  | - |
|  |  |  | 0.041 | 2655 | missense variant | c.691C>T,p.Arg231Cys | 20 | 57484607 | C | T |  |  | D | D | D | H | D | D | D | D | D | D |  |  |  |
|  |  |  | 0.021 | 2727 | splice site variant | c.840-3T>C | 20 | 57485003 | T | C | 3.2932E-05 | 0 | - | - | - | - | - | - | - | - | - | - |  |  |  |
|  |  |  | 0.024 | 3578 | missense variant | c.956C>T,p.Thr319Ile | 20 | 57485122 | C | T |  |  | D | U | D | M | D | D | D | D | D | D |  |  |  |
|  |  |  | 0.023 | 3787 | intron variant | c.971-9T>C | 20 | 57485380 | T | C | 0.00025192 | 0.008 | - | - | - | - | - | - | - | - | - | - |  |  |  |
|  |  |  | 0.055 | 3816 | synonymous variant | c.978C>T,p.Pro326= | 20 | 57485396 | C | T |  |  | - | - | - | - | - | - | - | - | - | - |  |  |  |
|  |  |  | 0.102 | 1241 | missense variant | c.1067G>A,p.Arg356His | 20 | 57485766 | G | A |  |  | T | D | D | M | D | D | D | D | D | D |  |  |  |
|  |  |  | 0.062 | 1241 | missense variant | c.1089C>A,p.Phe363Leu | 20 | 57485788 | C | A |  |  | D | D | D | M | D | D | D | D | D | D |  |  |  |
|  |  |  | 0.025 | 1244 | synonymous variant | c.1098T>C,p.Ala366= | 20 | 57485797 | T | C |  |  | - | - | - | - | - | - | - | - | - | - |  |  |  |
|  |  | Resection (relapsed) | 0.000 | 12676 |  | (c.601C>T,p.Arg201Cys) |  |  |  |  |  |  |  |  |  |  |  |  |  |  |  |  | - | - (0.02) | - |
|  |  |  | 0.000 | 12672 |  | (c.602G>A,p.Arg201His) |  |  |  |  |  |  |  |  |  |  |  |  |  |  |  |  | - |  | - |
|  |  | Normal (relapsed) | 0.000 | 9049 |  | (c.601C>T,p.Arg201Cys) |  |  |  |  |  |  |  |  |  |  |  |  |  |  |  |  |  |  |  |
|  |  |  | 0.000 | 9049 |  | (c.602G>A,p.Arg201His) |  |  |  |  |  |  |  |  |  |  |  |  |  |  |  |  |  |  |  |
| 14 | LGMFS | Biopsy | 0.020 | 2306 | synonymous variant | c.366C>T,p.Pro122= | 20 | 57478780 | C | T |  |  | - | - | - | - | - | - | - | - | - | - |  |  |  |
|  |  |  | 0.018 | 1991 | missense variant | c.367G>C,p.Glu123Gln | 20 | 57478781 | G | C |  |  | - | N | D | L | D | N | T | T | D | D |  |  |  |
|  |  |  | 0.022 | 1849 | synonymous variant | c.546C>T,p.Ile182= | 20 | 57484232 | C | T |  |  | - | - | - | - | - | - | - | - | - | - |  |  |  |
|  |  |  | 0.024 | 1847 | synonymous variant | c.576G>A,p.Pro192= | 20 | 57484262 | G | A |  |  | - | - | - | - | - | - | - | - | - | - |  |  |  |
|  |  |  | 0.000 | 2132 |  | (c.601C>T,p.Arg201Cys) |  |  |  |  |  |  |  |  |  |  |  |  |  |  |  |  | - | - (0.04) | - |
|  |  |  | 0.000 | 2132 |  | (c.602G>A,p.Arg201His) |  |  |  |  |  |  |  |  |  |  |  |  |  |  |  |  | - |  | - |
|  |  |  | 0.020 | 1767 | missense variant | c.674G>T,p.Gly225Val | 20 | 57484590 | G | T |  |  | D | D | D | H | D | D | D | D | D | D |  |  |  |
|  |  |  | 0.025 | 1773 | missense variant | c.685G>A,p.Asp229Asn | 20 | 57484601 | G | A |  |  | T | D | D | L | D | D | D | D | D | D |  |  |  |
|  |  |  | 0.051 | 2257 | splice site variant | c.1038+7C>T | 20 | 57485463 | C | T | 5.3223E-05 | 0 | - | - | - | - | - | - | - | - | - | - |  |  |  |
|  |  |  | 0.040 | 2005 | splice site variant | c.1039-5C>T | 20 | 57485733 | C | T | 0.00013571 | 0.02 | - | - | - | - | - | - | - | - | - | - |  |  |  |
|  |  |  | 0.029 | 2015 | missense variant | c.1067G>A,p.Arg356His | 20 | 57485766 | G | A |  |  | T | D | D | M | D | D | D | D | D | D |  |  |  |
|  |  |  | 0.037 | 2015 | missense variant | c.1117C>T,p.Arg373Cys | 20 | 57485816 | C | T |  |  | D | D | D | H | D | D | D | D | D | D |  |  |  |
|  |  |  | 0.021 | 2010 | missense variant | c.1139G>A,p.Arg380His | 20 | 57485838 | G | A |  |  | T | D | D | M | D | D | D | D | D | D |  |  |  |
|  |  | Resection | 0.018 | 727 | missense variant | c.188T>C,p.Leu63Pro | 20 | 57470715 | T | C |  |  | D | D | D | H | D | D | D | D | D | D |  |  |  |
|  |  |  | 0.024 | 668 | missense variant | c.280G>A,p.Asp94Asn | 20 | 57478608 | G | A |  |  | - | D | D | L | D | D | T | T | D | D |  |  |  |
|  |  |  | 0.069 | 1339 | missense variant | c.494G>A,p.Arg165His | 20 | 57480499 | G | A |  |  | D | D | D | M | D | D | D | D | D | D |  |  |  |
|  |  |  | 0.000 | 541 |  | (c.601C>T,p.Arg201Cys) |  |  |  |  |  |  |  |  |  |  |  |  |  |  |  |  | - | - (0.02) | - |
|  |  |  | 0.000 | 540 |  | (c.602G>A,p.Arg201His) |  |  |  |  |  |  |  |  |  |  |  |  |  |  |  |  | - |  | - |
|  |  |  | 0.169 | 581 | missense variant | c.793C>T,p.Arg265Cys | 20 | 57484813 | C | T |  |  | D | D | D | H | D | D | D | D | D | D |  |  |  |
|  |  |  | 0.146 | 581 | missense variant | c.794G>A,p.Arg265His | 20 | 57484814 | G | A |  |  | D | D | D | H | D | D | D | D | D | D |  |  |  |
|  |  |  | 0.082 | 1087 | missense variant | c.997C>T,p.Arg333Cys | 20 | 57485415 | C | T |  |  | D | D | D | L | D | D | D | D | D | D |  |  |  |
|  |  |  | 0.069 | 1085 | nonsense variant | c.1024C>T,p.Arg342* | 20 | 57485442 | C | T |  |  | - | D | D | - | - | - | - | - | - | D |  |  |  |
|  |  |  | 0.132 | 387 | splice site variant | c.1039-4G>A | 20 | 57485734 | G | A | 7.7034E-05 | 0.008 | - | - | - | - | - | - | - | - | - | - |  |  |  |
| 15 | LGMFS | Biopsy | 0.000 | 120 |  | (c.601C>T,p.Arg201Cys) |  |  |  |  |  |  |  |  |  |  |  |  |  |  |  |  | - | - (0.02) | - |
|  |  |  | 0.000 | 120 |  | (c.602G>A,p.Arg201His) |  |  |  |  |  |  |  |  |  |  |  |  |  |  |  |  | - |  | - |
|  |  | Resection | 0.030 | 3896 | missense variant | c.18C>A,p.Asn6Lys | 20 | 57466799 | C | A |  |  | T | - | D | L | D | N | D | D | D | D |  |  |  |
|  |  |  | 0.050 | 3912 | missense variant | c.28G>A,p.Glu10Lys | 20 | 57466809 | G | A |  |  | T | - | D | N | D | N | T | T | D | D |  |  |  |
|  |  |  | 0.053 | 3926 | missense variant | c.56A>G,p.Gln19Arg | 20 | 57466837 | A | G |  |  | T | - | D | L | D | N | T | T | D | D |  |  |  |
|  |  |  | 0.045 | 3924 | missense variant | c.79G>A,p.Glu27Lys | 20 | 57466860 | G | A |  |  | D | - | D | M | D | N | D | D | D | D |  |  |  |
|  |  |  | 0.029 | 9174 | missense variant | c.478C>T,p.Arg160Cys | 20 | 57480483 | C | T |  |  | D | N | D | L | D | D | D | D | D | D |  |  |  |
|  |  |  | 0.046 | 4363 | missense variant | c.595C>T,p.Arg199Cys | 20 | 57484414 | C | T |  |  | D | D | D | H | D | D | D | D | D | D |  |  |  |
|  |  |  | 0.000 | 4363 |  | (c.601C>T,p.Arg201Cys) |  |  |  |  |  |  |  |  |  |  |  |  |  |  |  |  | - | - (0.03) | - |
|  |  |  | 0.000 | 4362 |  | (c.602G>A,p.Arg201His) |  |  |  |  |  |  |  |  |  |  |  |  |  |  |  |  | - |  | - |
|  |  |  | 0.031 | 1559 | missense variant | c.664T>C,p.Phe222Leu | 20 | 57484580 | T | C |  |  | D | D | D | M | D | D | D | D | D | D |  |  |  |
|  |  |  | 0.160 | 1563 | missense variant | c.694C>T,p.Arg232Cys | 20 | 57484610 | C | T |  |  | D | D | D | H | D | D | D | D | D | D |  |  |  |
|  |  |  | 0.158 | 1392 | intron variant | c.840-11C>T | 20 | 57484995 | C | T | 1.3687E-05 | 0 | - | - | - | - | - | - | - | - | - | - |  |  |  |
|  |  |  | 0.065 | 5031 | missense variant | c.985G>A,p.Gly329Arg | 20 | 57485403 | G | A |  |  | D | N | D | H | D | D | D | D | D | D |  |  |  |
|  |  |  | 0.041 | 5019 | missense variant | c.1007G>A,p.Arg336Gln | 20 | 57485425 | G | A |  |  | T | D | D | M | D | D | D | D | D | D |  |  |  |
|  |  |  | 0.047 | 2051 | splice site variant | c.1038+4A>G | 20 | 57485460 | A | G | 0.99512727 | 0.924 | - | - | - | - | - | - | - | - | - | - |  |  |  |
|  |  | Normal | 0.000 | 5855 |  | (c.601C>T,p.Arg201Cys) |  |  |  |  |  |  |  |  |  |  |  |  |  |  |  |  |  |  |  |
|  |  |  | 0.000 | 5854 |  | (c.602G>A,p.Arg201His) |  |  |  |  |  |  |  |  |  |  |  |  |  |  |  |  |  |  |  |
| 16 | LGMFS | Biopsy | 0.000 | 40 |  | (c.601C>T,p.Arg201Cys) |  |  |  |  |  |  |  |  |  |  |  |  |  |  |  |  | - | - (0.02) | - |
|  |  |  | 0.000 | 40 |  | (c.602G>A,p.Arg201His) |  |  |  |  |  |  |  |  |  |  |  |  |  |  |  |  | - |  | - |
|  |  | Resection | 0.000 | 9183 |  | (c.601C>T,p.Arg201Cys) |  |  |  |  |  |  |  |  |  |  |  |  |  |  |  |  | - | - (0.02) | - |
|  |  |  | 0.000 | 9180 |  | (c.602G>A,p.Arg201His) |  |  |  |  |  |  |  |  |  |  |  |  |  |  |  |  | - |  | - |
| 17 | LGMFS | Biopsy | 0.000 | 3091 |  | (c.601C>T,p.Arg201Cys) |  |  |  |  |  |  |  |  |  |  |  |  |  |  |  |  | - | - (0.02) | - |
|  |  |  | 0.000 | 3091 |  | (c.602G>A,p.Arg201His) |  |  |  |  |  |  |  |  |  |  |  |  |  |  |  |  | - |  | - |
|  |  | Resection | 0.021 | 4059 | missense variant | c.253G>A,p.Asp85Asn | 20 | 57474036 | G | A |  |  | D | N | D | N | D | N | D | D | D | D |  |  |  |
|  |  |  | 0.035 | 3038 | synonymous variant | c.348C>T,p.Pro116= | 20 | 57478762 | C | T |  |  | - | - | - | - | - | - | - | - | - | - |  |  |  |
|  |  |  | 0.022 | 3032 | synonymous variant | c.366C>T,p.Pro122= | 20 | 57478780 | C | T |  |  | - | - | - | - | - | - | - | - | - | - |  |  |  |
|  |  |  | 0.032 | 4283 | synonymous variant | c.576G>A,p.Pro192= | 20 | 57484262 | G | A |  |  | - | - | - | - | - | - | - | - | - | - |  |  |  |
|  |  |  | 0.000 | 4504 |  | (c.601C>T,p.Arg201Cys) |  |  |  |  |  |  |  |  |  |  |  |  |  |  |  |  | - | - (0.03) | - |
|  |  |  | 0.000 | 4503 |  | (c.602G>A,p.Arg201His) |  |  |  |  |  |  |  |  |  |  |  |  |  |  |  |  | - |  | - |
| 18 |  | Resection | 0.029 | 1665 | missense variant | c.74A>G,p.Lys25Arg | 20 | 57466855 | A | G |  |  | T | - | D | L | D | N | T | T | D | D |  |  |  |
|  |  |  | 0.023 | 575 | missense variant | c.395T>C,p.Leu132Pro | 20 | 57478809 | T | C |  |  | - | D | D | M | D | D | D | D | D | D |  |  |  |
|  |  |  | 0.044 | 498 | missense variant | c.425T>C,p.Phe142Ser | 20 | 57478839 | T | C |  |  | - | D | D | M | D | D | D | D | D | D |  |  |  |
|  |  |  | 0.084 | 355 | missense variant (splice site variant) | c.585G>T,p.Gln195His | 20 | 57484271 | G | T | 0.99998873 | 0.994 | D | D | D | H | D | D | D | D | - | D |  |  |  |
|  |  |  | 0.000 | 354 |  | (c.601C>T,p.Arg201Cys) |  |  |  |  |  |  |  |  |  |  |  |  |  |  |  |  | - | - (0.04) | - |
|  |  |  | 0.000 | 354 |  | (c.602G>A,p.Arg201His) |  |  |  |  |  |  |  |  |  |  |  |  |  |  |  |  | - |  | - |
|  |  |  | 0.066 | 518 | missense variant | c.831G>T,p.Trp277Cys | 20 | 57484851 | G | T |  |  | T | D | D | N | D | D | T | D | D | D |  |  |  |
|  |  |  | 0.245 | 359 | synonymous variant | c.891C>T,p.Leu297= | 20 | 57485057 | C | T |  |  | - | - | - | - | - | - | - | - | - | - |  |  |  |
|  |  | Normal | 0.000 | 3741 |  | c.601C>T,p.Arg201Cys |  |  |  |  |  |  |  |  |  |  |  |  |  |  |  |  |  |  |  |
|  |  |  | 0.000 | 3741 |  | c.602G>A,p.Arg201His |  |  |  |  |  |  |  |  |  |  |  |  |  |  |  |  |  |  |  |
|  |  |  | 0.024 | 3017 | synonymous variant | c.891C>T,p.Leu297= | 20 | 57485057 | C | T |  |  | - | - | - | - | - | - | - | - | - | - |  |  |  |
| 19 | LGMFS | Resection | 0.000 | 11688 |  | (c.601C>T,p.Arg201Cys) |  |  |  |  |  |  |  |  |  |  |  |  |  |  |  |  | - | - (0.02) | - |
|  |  |  | 0.000 | 11687 |  | (c.602G>A,p.Arg201His) |  |  |  |  |  |  |  |  |  |  |  |  |  |  |  |  | - |  | - |

IM, Intramuscular myxoma; LGMFS, Low-grade myxofibrosarcoma; Normal, Normal tissue around the tumor; PCR-DS, PCR-direct sequencing; Fragment signal analysis, Fragment signal analysis using restriction digestion and capillary electrophoresis after PCR combined with PNA clamping; SR, signal ratio; PCR-DS with PNA, PCR-direct sequencing combined with PNA clamping

SIFT/FATHMM/MetaSVM/MetaLR/M-CAP: D, Damaging; T, Tolerated.

LRT: D, Deleterious; N, Neutral; U, Unknown.

MutationTaster: A, Disease causing automatic; D, Disease causing; N, Polymorphism; P, Polymorphism automatic.

MutationAssessor: H, Predicted functional (High); M, Predicted functional (Medium); L, Predicted non-functional (Low); N, Predicted non-functional (Neutral).

PROVEAN/fathmm-MKL: D, Damaging; N, Neutral.
