# Supplementary material for: Molecular Characterization and Its Clinical Application of GNAS Variants in Intramuscular Myxoma
Source: Cancer Med. 2026 Mar 29;15(4):e71751. doi: 10.1002/cam4.71751 (PMC13140542; doi:10.1002/cam4.71751)
Supplement: Supplementary file 2 — Data S1: Supporting Information. [file CAM4-15-e71751-s002.docx]

**Supplementary Methods**

*S1.* *A custom NGS panel-based analysis for GNAS*

An NGS panel for *GNAS* optimized for FFPE samples was designed using Ion AmpliSeq Designer (https://ampliseq.com/browse.action). Uracil DNA Glycosylase (UDG) (Thermo Fisher Scientific, Waltham, MA, USA) treatment was performed before library preparation to remove uracil bases from the template DNA produced by cytosine deamination. Library preparation was performed on Ion Chef (Thermo Fisher Scientific) using an Ion AmpliSeq Kit for Chef DL8 (Thermo Fisher Scientific). Sequencing was performed on an Ion Chef and Ion GeneStudio S5 (Thermo Fisher Scientific) using an Ion 510 & Ion 520 & Ion 530 Kit-Chef and an Ion 510 Chip Kit (Thermo Fisher Scientific). Sequencing data were mapped using Torrent Suite software 5.8 (Thermo Fisher Scientific) to the human genome hg19. Variants were detected using the Torrent Variant Caller plug-in, with default settings for somatic low-stringency parameters. A HotSpot file that defines R201C (chr20:57484420) and R201H (chr20:57484421) in *GNAS* and enabled the identification of whether these variants were present. The detected variants were annotated using SnpEff and SnpSift (http://snpeff.sourceforge.net) using the processed vcf file of dbNSFP3.4c and dbscSNV1.1 (https://sites.google.com/site/jpopgen/dbNSFP). Germline variants, intronic variants (>−12 or >+8 from exon-intron boundaries), and variants in untranslated regions were excluded. These variants were described using the NM_000516.5 transcript reference sequence, and the variant nomenclature was in accordance with the recommendations of the Human Genome Variation Society.

*S2. Genetic screening for R201 variants in GNAS*

*S2.1 PCR-direct sequencing analysis*

PCR was performed using tagged primers for sequencing targeting *GNAS* exon 8: forward: 5'-CAGGAAACAGCTATGACCACGGCGTTGGCTTTGGTGAGATCC-3,’ reverse: 5'-TAATACGACTCACTATAGGGTTGTCCACCTGGAACTTGGT -3' (underlined text denotes tag sequence). The PCR product was confirmed by agarose gel electrophoresis and a band of the desired size was extracted using the QIAEX II Gel extraction kit (Qiagen, Hilden, Germany). Sequencing reactions were then performed using the BigDye™ Terminator v3.1 Cycle Sequencing Kit (Applied Biosystems, Foster City, CA, USA), and the base sequence was analyzed and determined using a 3500 Genetic Analyzer (Applied Biosystems) and DNA Sequencing Analysis software v5.1 (Applied Biosystems).

*S2.2 Fragment signal analysis by restriction digestion and capillary electrophoresis after PCR combined with PNA clamping*

The PCR reaction using PNA clamping used 6-FAM fluorescent labelling primers at the 5' end of the forward and reverse primers, as shown in section S2.1. and PNA: N-GCTGCCGTGTCCT-C (Panagene Inc., Daejeon, Korea). The PCR reaction mixture contained 6.9 ng (range: 0.5-45.8 ng; median) of genomic DNA, 1× Ampdirect™ Plus (Shimadzu Corporation, Kyoto, Japan), 1.5 units of BIOTAQ™ HS DNA polymerase (Bioline, London, UK), and 0.4 units of UDG Heat-labile (TOYOBO, Osaka, Japan). The final PNA concentration was 2.5 μmol/L, final primer concentration was 0.3 μmol/L, and reaction volume was 20 μL. The PCR reaction conditions were set as follows: initial denaturation at 95°C for 10 min, followed by 40 cycles of denaturation at 95°C for 15 s, annealing at 60°C for 30 s, and extension at 72°C for 45 s, with a final extension step at 72°C for 7 min. Additionally, an initial step of UDG incubation at 25°C for 5 min to remove uracil bases from the template DNA produced by cytosine deamination was included.

For restriction enzyme treatment of the PCR product, 2 μL of the PCR product was mixed with a reaction solution containing 1 unit of BceAⅠ (New England BioBabs, Hitchin, UK) and the reaction was performed at 37℃ for 1 h. The reaction was subsequently carried out at 65℃ for 20 min due to enzyme inactivation. The restricted enzyme treatment reaction solution was diluted with purified water, mixed with HiDi formamide (Applied Biosystems) containing GeneScan^TM^ 350 ROX^TM^ Dye Size Standard (Applied Biosystems), and heat-denatured. Capillary electrophoresis was performed on a 3500 Genetic Analyzer (Applied Biosystems). GeneMapper software (Applied Biosystems) was used for the analysis. The peak observed at 81 bp was defined as the wild-type allele peak, the peak observed at 109 bp was defined as the variant allele peak, and the signal ratio (SR) was calculated as the height of the variant allele peak/height of the wild-type allele peak.

The schema of the assay design is shown in Fig. 3

*S2.3 PCR-direct sequencing analysis combined with PNA clamping*

Sequence analysis was performed in the same manner as described in section S2.1, using the PCR product obtained by the method described in section S2.2.

*S2.4 Sensitivity study*

To assess variant detection sensitivity, we cloned the wild-type sequence of *GNAS* exon 8 using the TA Cloning Kit with pCR2.1 Vector (Invitrogen, Waltham, MA, USA) and generated recombinant plasmids. After transforming One Shot™ INVαF' Chemically Competent E. coli with the recombinant plasmids and culturing, plasmid DNA was extracted using the QIAprep Spin Miniprep Kit (Qiagen, Hilden, Germany). Additionally, variants were introduced into the extracted wild-type sequence plasmid DNA using the PrimeSTAR® Mutagenesis Basal Kit (Takara Bio Inc., Shiga, Japan), resulting in plasmids with mutant sequences (c.601C>T,p.R201C; c.602G>A,p.R201H). DNA quantity was determined using the copy number of the constructed plasmids. Mutant sequence plasmids were diluted using wild-type sequence plasmids to create a dilution series of 20, 10, 5, 2, and 1%. Fragment signal analysis using restriction digestion and capillary electrophoresis after PCR combined with PNA clamping was performed using mutant sequence plasmids at each concentration produced.
